# Ammonia Binding to the Oxygen-Evolving Complex Probed by High-Energy Resolution Fluorescence Detected X-Ray Absorption Spectroscopy

**DOI:** 10.1021/acs.jpcb.5c00269

**Published:** 2025-04-03

**Authors:** Maria Chrysina, Maria Drosou, Dimitrios A. Pantazis, Serena DeBeer

**Affiliations:** † Max Planck Institute for Chemical Energy Conversion, Stiftstr. 34-36, Mülheim an der Ruhr 45470, Germany; ‡ Institute of Nanoscience & Nanotechnology, NCSR “Demokritos”, Athens 15310, Greece; § Max-Planck-Institut für Kohlenforschung, Kaiser-Wilhelm-Platz 1, Mülheim an der Ruhr 45470, Germany

## Abstract

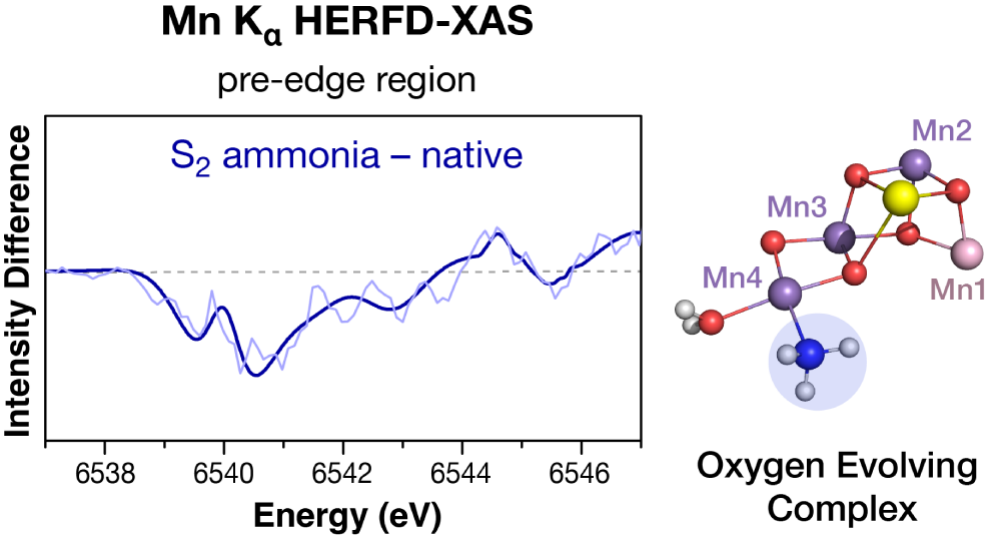

The insertion pathways and binding sites of substrate water molecules at the catalytic Mn_4_CaO_5_ cluster of the oxygen-evolving complex (OEC) in photosystem II (PSII) remain a fundamentally unresolved question toward understanding biological water oxidation. To address this question, small molecules have been employed as “water analogues” to probe substrate binding to the OEC. In this context, the binding of ammonia has been extensively investigated and discussed using spectroscopic, structural, and quantum chemical methods, but a definitive answer regarding the ammonia binding site has not yet been achieved. Herein, we present high-energy resolution fluorescence detected (HERFD) Mn K-edge X-ray absorption spectroscopy (XAS) in ammonia-treated S_2_ state samples of the OEC. Pre-edge features were correlated with possible structural models with the aid of quantum chemical calculations. The comparison of calculated and experimental difference spectra between the native and ammonia-treated samples allows us to evaluate different modes of ammonia interaction with the OEC. The combined spectroscopic and theoretical investigation suggests the substitution of the terminal water ligand W2 on Mn4 as the most plausible ammonia binding mode, followed closely by the substitution of the second terminal water ligand (W1), and the coordination of ammonia on Mn1 as a sixth ligand. Our results are in line with the leading interpretations of other spectroscopic and kinetic studies, converging on the conclusion that the Mn4 ion is either the most accessible or the strongest binding site for substrate analogues.

## 1 Introduction

**Figure 1:**
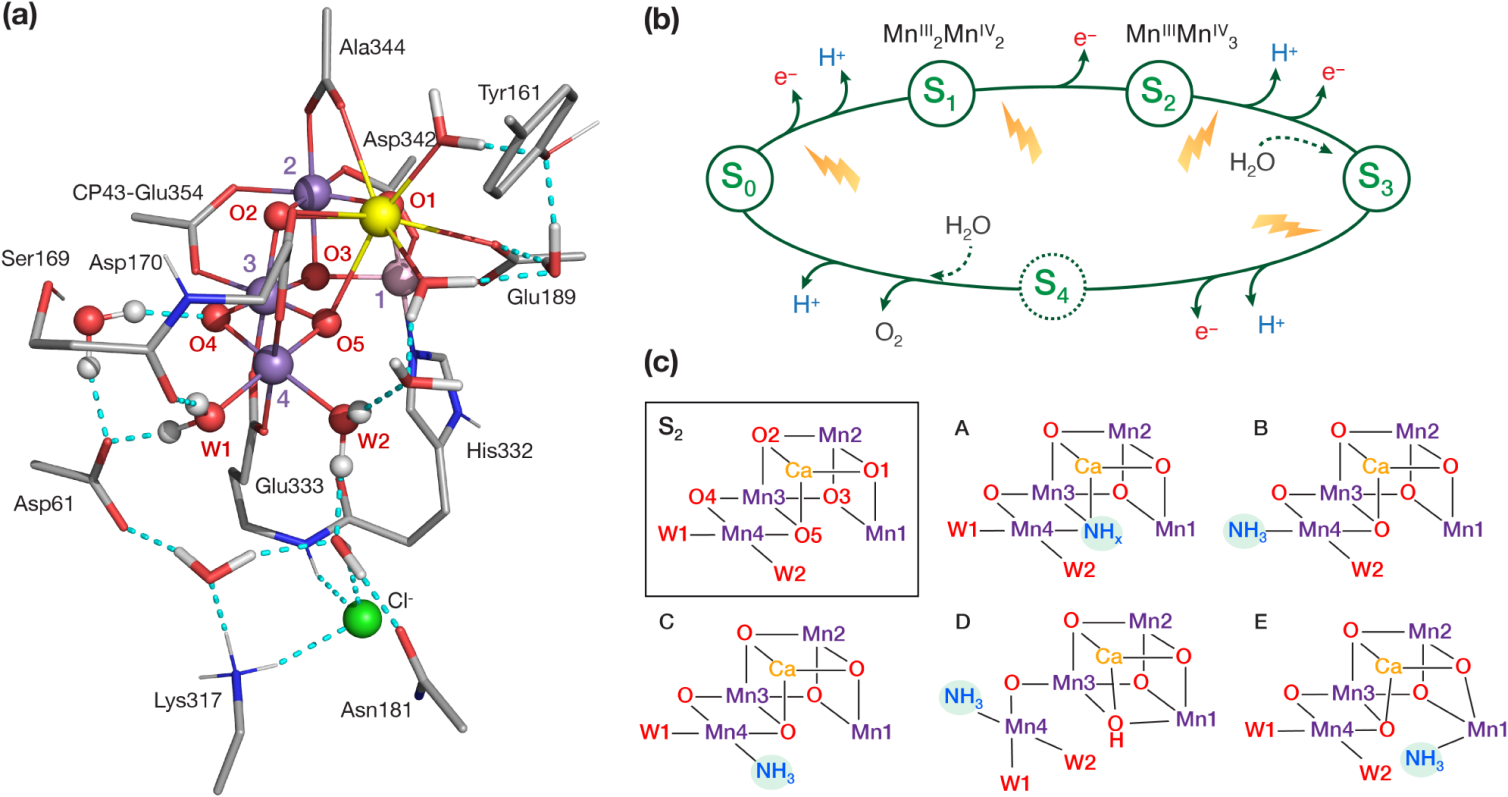
(a) The oxygen-evolving complex of PSII and part of its protein environment; (b) the Kok–Joliot cycle of water oxidation; (c) possible ammonia binding modes (A–E) discussed in the literature.

Biological dioxygen generation from water is carried out by the oxygen-evolving complex (OEC) in photosystem II (PSII) in plants, algae, and cyanobacteria.^1−5^ The OEC consists of a protein-embedded Mn_4_CaO_5_ inorganic core, which forms a near-cuboidal Mn_3_CaO_4_ unit connected to the fourth Mn ion (Mn4) by two bridging oxo groups (O4 and O5, Figure 1a).^6,7^ The complex cycles through five intermediate states S*_i_* (*i* = 0–4) to store the four oxidizing equivalents required for the four-electron oxidation of water to dioxygen (Figure 1b).^8,9^ The two substrate water molecules bind at different states of the catalytic cycle, one during or after the S_2_ → S_3_ transition,^10−21^ and another during the S_4_ → S_0_ dioxygen formation and release step.^22−29^ Three water channels that begin from the OEC near O1, O4, and the proximal Cl (Figure 1a), respectively, and extend toward the aqueous environment of PSII, mediate the transfer of substrate water molecules and the removal of protons.^30−39^ Given the difficulty in identifying the atomic details of water delivery and binding,^3,37,40−43^ small molecules like ammonia have long been used as water analogues to probe substrate binding to the OEC.^44−55^

Two modes of ammonia binding on the OEC have been determined.^56,57^ One is chloride-dependent and inhibits O_2_ evolution,^56−59^ whereas the other binding mode is independent of the presence of chloride, and it slows down but does not inhibit O_2_ evolution.^56,57,60−63^ In the second binding mode, ammonia is believed to bind covalently to the cluster. This is based on experimental observations from electron paramagnetic resonance (EPR) spectroscopy,^61,64^ electron–nuclear double resonance (ENDOR) spectroscopy (^55^Mn hyperfine coupling constants),^49,55^ electron spin echo envelope modulation (ESEEM),^49,50,61^ and electron–electron double-resonance–detected NMR (EDNMR),^47,49^ Fourier transform infrared (FTIR) difference spectroscopy,^65,66^ and kinetics studies.^67^ Nevertheless, none of these studies can pinpoint the precise mode of ammonia noninhibitory binding, which therefore remains contested. Identification of this binding site serves to elucidate the pathway for water delivery to the cluster and possibly correlates with the site of substrate binding.

Five proposed ammonia binding modes are shown in Figure 1c. Binding mode A, originally suggested based on ^14^N hyperfine anisotropy measured from ESEEM,^61^ and later supported by interpretations of ammonia inhibition kinetic studies^67^ and FTIR experiments,^66^ involves O5 oxo-bridge substitution by an imido (NH^2–^), amido (NH_2_^–^), or nitrido (N^3–^) bridge.^68^ In binding modes B and C, ammonia binds to Mn4 as a terminal ligand, replacing W1 and W2, respectively. Terminal ligand substitution has been supported by magnetic spectroscopy^47,49^ and quantum chemical studies.^69−71^ If ammonia remains coordinated to the cluster during the S_3_ → S_0_ transition, noninhibitory binding replacing one of the terminal W1 or W2 ligands would preclude the specific ligand from being a substrate in the subsequent O–O bond formation step. In binding modes D^72^ and E,^73^ ammonia binds to Mn4 and Mn1, respectively, in closed and open-cubane conformations of the cluster, as an additional sixth terminal ligand. Crystallographic studies^74^ and extended X-ray absorption fine structure (EXAFS)^75^ cannot be used as a basis to distinguish between models that represent the different binding modes, probably because the structural changes induced by ammonia binding to the OEC cluster are too small.

However, important information might be contained in the pre-edge region of the Mn K-edge X-ray absorption spectrum, even for species having the same Mn oxidation states,^76,77^ if this is sufficiently resolved. The Mn K-edge XAS arise from excitations of the core (1s) electrons of Mn into unoccupied molecular orbitals. Dipole allowed 1s → 4p excitations give rise to the intense region known as the edge. The energy of the edge correlates with the oxidation states of the Mn ions and has been used to probe the total oxidation state of the Mn ions of the Mn_4_CaO_5_ cluster.^78−80^ The lower energy pre-edge region is dominated by 1s → 3d excitations. Even though these transitions are formally dipole-forbidden, they gain intensity through the introduction of dipole character via the mixing of metal p character into the d manifold.^81^ Hence, the pre-edge region contains information on the local electronic structure of the metal ion, as well as its geometry, coordination number, and ligand covalency.

A highly resolved description of the Mn K pre-edge region for all S-states of the OEC, obtained using high-energy resolution fluorescence detected (HERFD) X-ray absorption spectroscopy (XAS),^82−84^ was recently used in combination with quantum chemical calculations to resolve the nature of oxidation events in all spectroscopically observable catalytic intermediates of the OEC.^77^ Herein, we employ Mn HERFD XAS combined with quantum chemical calculations to discriminate between the various suggested modes of ammonia binding on the S_2_ state of the OEC by comparing the calculated Mn spectra of selected structural models that represent all proposed ammonia binding modes with experimental data. Our results indicate that models in which ammonia replaces one of the two terminal water ligands on Mn4 show the highest alignment with experimental data, followed by Mn1 addition, while they disfavor substitution of a bridging μ-oxo ligand or the addition of ammonia as a sixth ligand on Mn4 in the closed cubane conformation of the cluster.

## 2 Methods

### 2.1 Experimental Details

#### 2.1.1 Photosystem II Sample Preparation

Photosystem II was purified from the thermophilic cyanobacterium *Thermosynechococcus vestitus* according to Kuhl et al.^85^ The final buffer contains 500 mM mannitol, 40 mM MES (pH = 6.5), 10 mM CaCl_2_, 10 mM MgCl_2_, 0.03% v/v *n*-dodecyl β-d-maltoside. Ammonia treatment: photosystem II preparations were washed with buffer-free solution (500 mM mannitol, 10 mM CaCl_2_, 10 mM MgCl_2_) until the MES concentration was <1 mM. Solution of 1 M NH_4_Cl in 1 M HEPES (pH = 7.6) was added at a ratio of 1:10 v/v to the sample, i.e., giving a final concentration of 100 mM, which equates to 2 mM NH_3_ in solution. Phenyl-para-benzoquinone (PPBQ) dissolved in dimethyl sulfoxide (DMSO) was added as an electron acceptor at a final concentration of 0.5 mM. Photosystem II (1.5 mM Mn, 25 μL) was loaded in POM frames sealed with Mylar tape. S_2_ samples were 1-flash illuminated using a Nd:YAG laser (532 nm, 10 ns pulse of 500 mJ) at room temperature and frozen in liquid N_2_. EPR measurements were performed on all samples to ensure the high quality and incorporation of ammonia in the ammonia-treated samples. A Bruker E500 spectrometer equipped with a Bruker ER 4116DM resonator and an Oxford Instruments ESR 900 cryostat was used for the EPR measurements.

#### 2.1.2 HERFD Measurements

Mn K_α_ HERFD measurements were performed at beamline ID26 of the European Synchrotron Radiation Facility (ESRF). The current of the Extremely Brilliant Source (EBS) storage ring was 90 mA (16 bunch mode). The incident beam was monochromatized using a pair of cryogenically cooled Si(311) crystals, giving an incident energy resolution of ∼0.2 eV. The flux is estimated to be ∼10^12^ photons/s in a 200 × 100 μm beam spot. The energy of the incident beam was calibrated by setting the first inflection point of the XAS spectrum of a Fe foil to 7111.2 eV. K_α_-HERFD were collected using a Johann-type XES spectrometer with five Ge(111) crystals and an energy-dispersive Si drift detector. The spectrometer resolution at the elastic peak was ∼0.7 eV. The sample temperature during measurements was poised at 20 K using a liquid He-cooled flow cryostat. To establish the highest X-ray dose that does not damage the sample, time scans were performed at 6550 eV, which was proved to be a sensitive probe for damage. Aluminum filters were used to attenuate the beam at about 11%. Once the dwell time was estimated by the time scans at the edge position (6550 eV), X-ray absorption near edge spectroscopy (XANES) was performed using the respective dose and a lower dose to ensure that the two spectra overlap. Finally, the dose used for the S_1_ state (0 flashes) was 3.9 × 10^7^ photons/μm^2^ and 1.9 × 10^7^ photons/μm^2^ for the S_2_ state (1 flash). The K_α_ HERFD XANES scans were collected in an energy range of 6535–6570 eV and the pre-edge region was measured at 6537–6547 eV, while long 6530–6800 eV scans were measured to facilitate proper normalization. The energy step was, in all cases, 0.1 eV. The XANES spectra were normalized by area according to the long scans; subsequently, the pre-edge region was normalized by area to the XANES spectra pre-edge region. The estimated errors introduced after normalization are 1–2%, which is negligible for the pre-edge intensities. For further analysis, high-resolution pre-edge region scans were used. The signal-to-noise ratio of the HERFD spectra is ∼20.

### 2.2 Computational Details

Cluster models of the OEC in the native S_2_ state and of possible ammonia-bound S_2_ state forms were constructed and optimized as described in previous work.^70^ Starting coordinates were obtained from the 5B66 crystallographic structure reported by Tanaka et al.^86^ The models include the inorganic Mn_4_CaO_5_ core, all first coordination sphere amino acids and water ligands (W1–W4), the second sphere amino acids Asp61, Tyr161, Gln165, Ser169, Asn181, Val185, Phe186, His190, Asn298, Lys317, His337, Leu343, and CP43-Arg357, 13 more crystallographic water molecules, and the proximal chloride Cl^–^ ion. In a previous study,^70^ 29 different ammonia-bound S_2_ state models were screened based on their magnetic and spectroscopic properties. Among this set, only 14 models have a predicted *S* = 1/2 ground state, consistent with the EPR of the ammonia-treated samples. Therefore, XAS calculations were performed in the present work only on these 14 models that satisfy the *S* = 1/2 ground state.

All computations were conducted using Orca 5.^87^ The XAS spectra were calculated as previously reported,^77,88−91^ via the TD-DFT method, employing the Tamm–Dancoff approximation.^92^ The TPSSh functional^93^ was selected for its proven accuracy in previous studies. Relativistic effects were considered throughout using the zeroth-order regular approximation (ZORA),^94−97^ along with the scalar-relativistically recontracted^98^ ZORA-def2-TZVP(-f) basis sets^99^ for all atoms except C and H for which the ZORA-def2-SVP basis sets were used. The conductor-like polarizable continuum model (CPCM) solvation model^100^ with dielectric constant ε = 6.0 was used to simulate the effect of the protein surrounding. To optimize computational efficiency, the resolution of identity (RI) and chain of spheres (RIJCOSX) approximations^101,102^ were employed along with the decontracted def2/J auxiliary basis sets.^103^ The XAS spectra for each Mn ion were computed separately considering 150 roots. The final spectra for each model were derived by summing the individual spectra of all four Mn ions. An energy shift of 35.3 eV and intensity scaling of 0.018 were applied to all models on the basis of optimal alignment with experimental data of the untreated S_2_ state. This serves to eliminate systematic errors intrinsic to the computational approach, a usual practice in TD-DFT studies of XAS pre-edge spectra,^77,88−91^ and to ensure common treatment of all models for comparing their corresponding spectroscopic features directly on the same basis.

Evaluation of the ammonia-bound OEC models was based on the difference spectra between the ammonia-treated and untreated samples. The degree of deviation of the calculated difference spectra of each ammonia-bound model from the experimental difference spectra was quantified using the root-mean-square deviation (RMSD) of the calculated difference spectra from the experimental difference spectra:
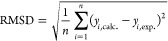
where *y*_*i*_,_calc._ and *y*_*i*_,_exp._ are the intensities of the calculated and experimental difference spectra, respectively, at 240 energy values in the energy region 6538–6544.5 eV.

## 3 Results and Discussion

### 3.1 Mn K_α_ Edge High Energy Resolution Fluorescence Detected X-Ray Absorption Spectroscopy

**Figure 2:**
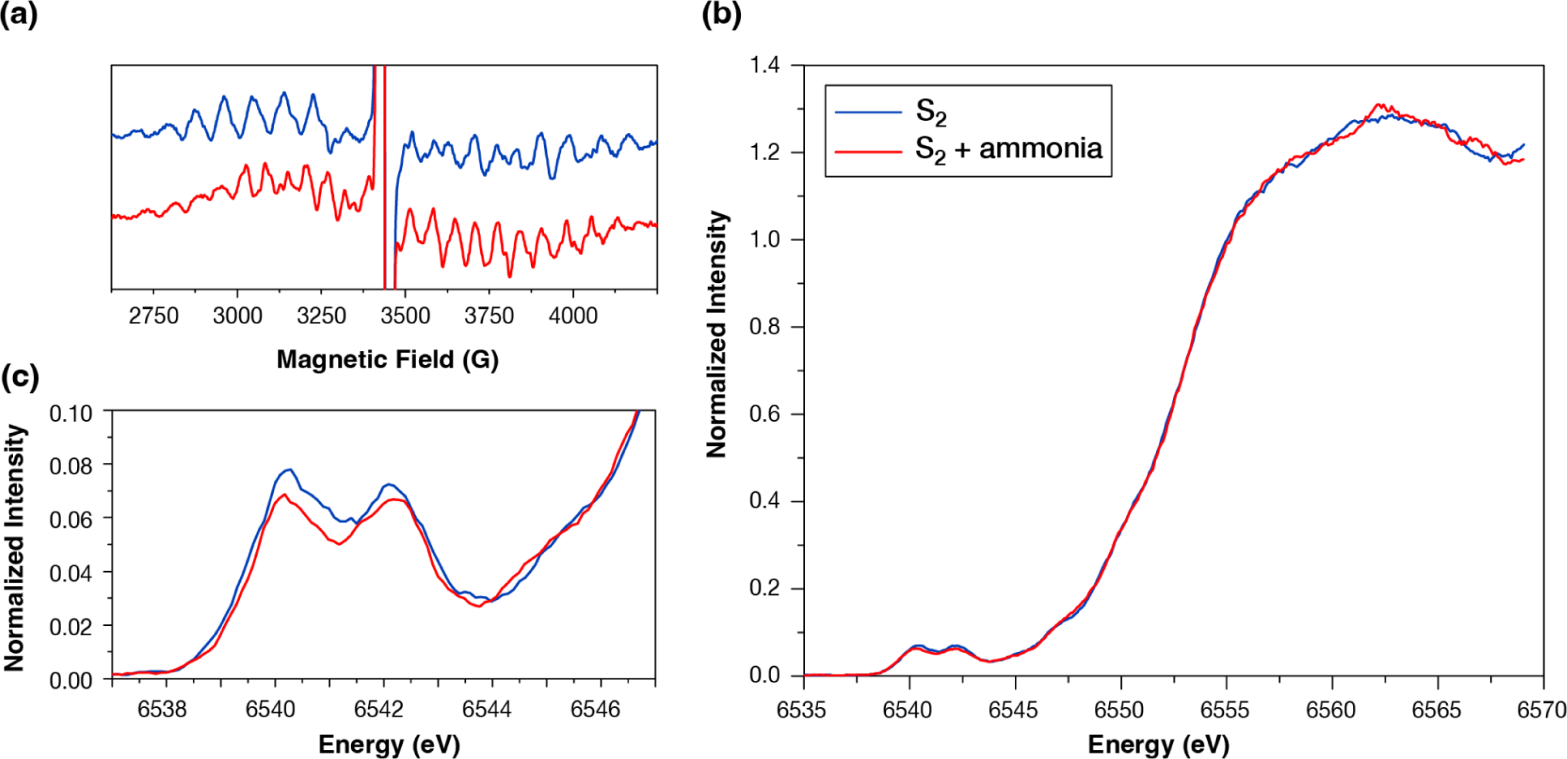
Comparison of EPR and Mn K-edge HERFD XAS spectra of the native (blue) and ammonia-treated (red) S_2_ state. (a) EPR multiline signal; EPR parameters: modulation frequency: 100 kHz, modulation amplitude: 7 G peak to peak, microwave frequency: 9.64 GHz, microwave power: 8 mW, sweep time: 100 s, sweep field: 5000 G, average of 4 scans, temperature: 10 K. (b) XANES region, and (c) Mn K_α_ HERFD pre-edge region. The energy grid of both XANES and pre-edge spectra is 0.1 eV. The pre-edge region is binned by a 5-point window, while the XANES region is binned by an 11-point window.

Dark-adapted and 1-flashed samples of untreated and ammonia-treated PSII isolated from *T. vestitus* were prepared and their Mn K_α_ HERFD spectra were measured. All samples were studied by EPR prior to the X-ray measurement. In the dark-adapted samples, there was no sign of Mn(II) that would indicate Mn release from Photosystem II due to poor sample quality. After 1 laser flash, the multiline (*g* ≈ 2) EPR signal of the S_2_ state^104^ was observed and its intensity was used as a probe of the S_2_ population in the samples. A small population (∼10%) of the 0-flashed HERFD spectrum was subtracted from the 1-flashed spectrum to acquire the pure S_2_ state spectra for the untreated and ammonia-treated photosystem II. The multiline EPR signals of the untreated and the ammonia-treated S_2_ samples are presented in Figure 2a. The quantitative incorporation of ammonia in the Mn_4_Ca cluster was verified based on the modification of the S_2_ multiline EPR signal.^60^

The Mn K_α_ HERFD absorption spectra of the untreated (native) and ammonia-treated PSII samples were measured at ID26 at the ESRF. The raw data of the XANES regions are shown in Figure S1 and a comparison of the raw and binned spectra is shown in Figure S2. Ammonia treatment does not induce perceptible changes in the S_1_ state spectra, consistent with past suggestions that in the S_1_ state ammonia does not interact with Mn.^61^ In contrast, the covalent binding of ammonia to a Mn ion of the OEC cluster is considered to be taking place during the S_1_ → S_2_ transition.^61,66^

The XANES region of the native S_2_ state compared to the ammonia-treated sample is shown in Figure 2b. It can be observed that there is no difference between the native and the ammonia-treated samples in the edge region, suggesting that there is no difference in the oxidation state of the Mn cluster or any major change in the local environment of the Mn ions upon ammonia binding. The effect of ammonia binding is observed in the pre-edge region, presented in Figure 2c. Upon ammonia binding to the S_2_ state, the absorption intensity of the pre-edge region decreases most prominently in the region between 6540–6542 eV and the decrease is less pronounced in the region between 6542–6543 eV. A polynomial spline was fitted to the rising edge and subtracted. Subsequently, three Voigt curves were fitted to the experimental spectra (Figure S3), with the parameters presented in Table S1.

**Figure 3:**
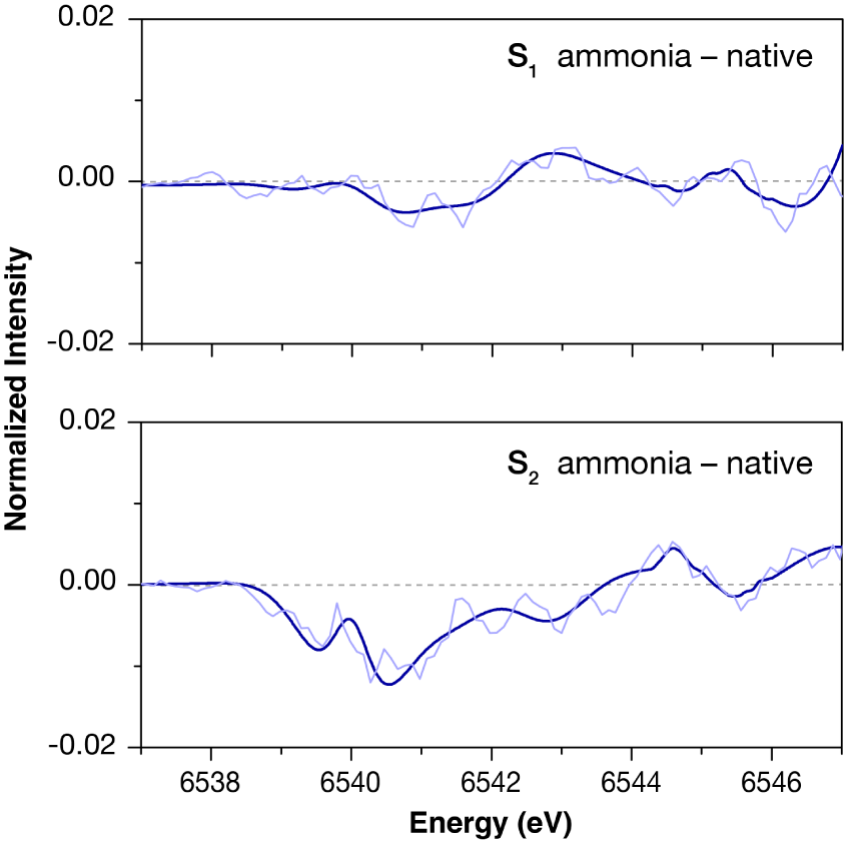
Difference spectra between ammonia-treated samples and the native S_1_ (top) and S_2_ (bottom) samples. The differences between the 5-point binned experimental spectra are presented as light lines and the differences between the fitted spectra as dark lines. The difference between ammonia and native for the S_2_ state is the subtraction of the spectra shown in Figure 2c.

Figure 3 shows the Mn K-edge XAS difference spectra between the native and the ammonia-treated S_1_ and S_2_ states in the pre-edge region. The binned and fitted spectra used to calculate the differences are presented in Figure S4, alongside the corresponding raw data. We use the differences between the fitted spectra as a basis for further analysis to avoid the amplification of noise-related artifacts that would result from subtracting the raw spectra. As shown in Figure 3, the intensity difference is near zero in the S_1_ state, whereas in the S_2_ state, the largest intensity differences are observed between 6539 and 6542 eV. Excitations in this region have been previously attributed to local excitations from the 1s to the 3d orbitals of Mn.^77^ Therefore, these differences indicate small geometry changes in the coordination sphere of one or more Mn ions of the cluster upon ammonia binding. In the next sections, we attempt to distinguish the ammonia binding modes that reproduce these changes with the aid of quantum chemical calculations.

### 3.2 Correlation with Structural Models

**Figure 4:**
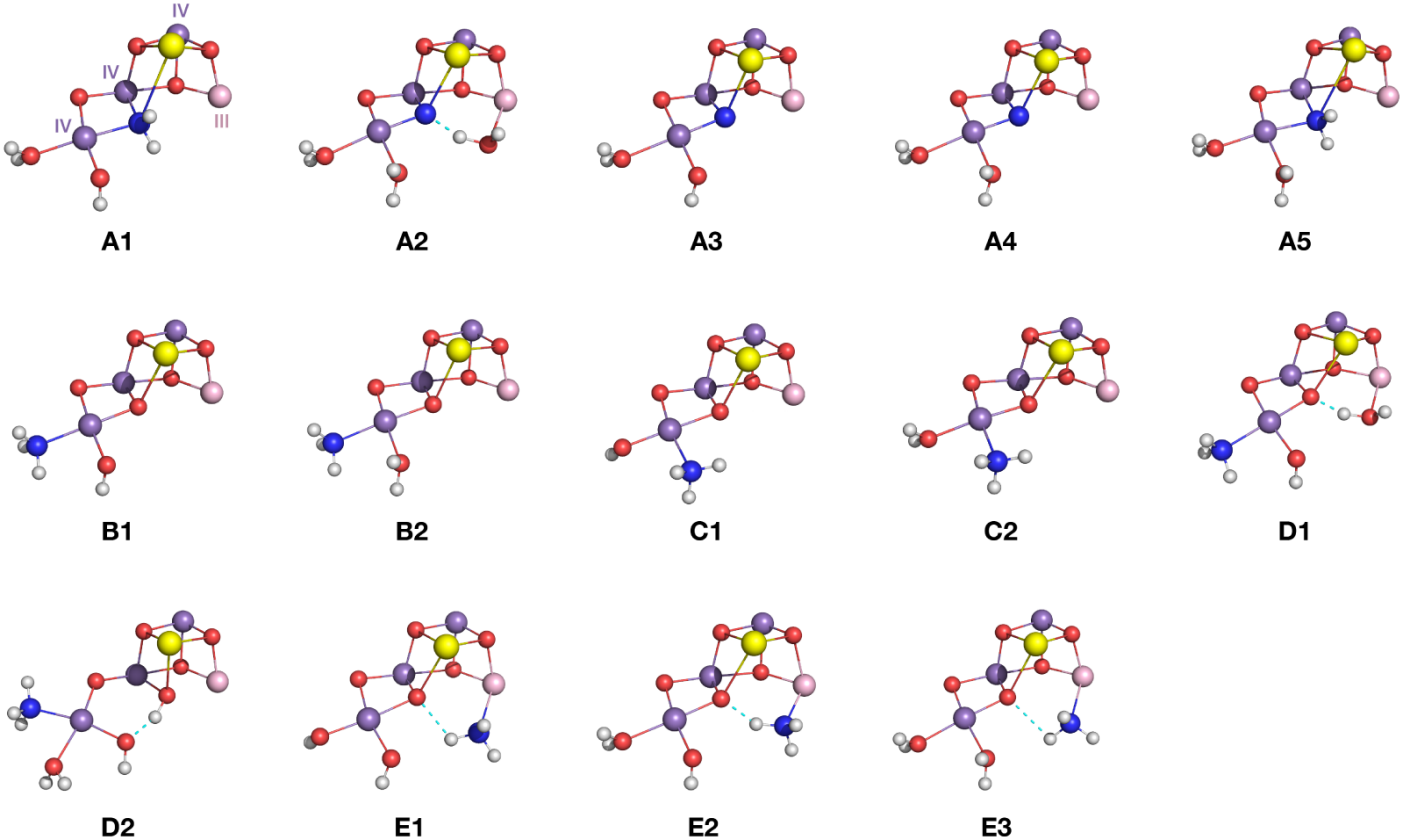
Structures of the inorganic cores of the ammonia-bound S_2_ state models. Mn(IV) ions are shown in purple, Mn(III) in pink, O atoms in red, N in blue, Ca^2+^ ions in yellow, and H atoms in white.

To determine what the pre-edge region of the Mn XAS spectra reveals about ammonia binding in the S_2_ state, we employed quantum chemical calculations. Building on our previous study, which focused on magnetic properties and energetics,^70^ we examined 14 ammonia-bound S_2_ state models (Figure 4) that represent variations of the proposed ammonia binding modes described in Figure 1c. Since geometry optimization is conducted independently of the property under investigation, we used the same models to maintain consistency in our assessment. We note that the calculated difference spectra between the S_1_ and S_2_ states, whose structural identity has been previously established,^77^ reproduce the experimental difference spectra well (Figure S5). In type A models, ammonia replaces the O5 μ-oxo bridge of the cluster generating an amido (NH_2_^–^) bridging ligand in **A1** and **A5**, and a nitrido bridge (N^3–^) in **A2**, **A3**, and **A4**. In type B and C models, ammonia substitutes the terminal Mn4 water ligands W1 and W2, respectively. Type D models feature ammonia as an additional ligand on Mn4 without dissociating a water molecule from the cluster. Finally, in type E models, ammonia coordinates as an additional ligand to the open coordination site of Mn1(III).

Given that the protonation states of the OEC are difficult to probe experimentally and there is no conclusive protonation state assignment either for the native or for the ammonia-bound S_2_ state,^52,105−107^ we examined variants with different protonation states for each type of model. We note that all models have the same valence distribution [Mn1, Mn2, Mn3, Mn4] = [III, IV, IV, IV]. Moreover, all models have a *S* = 1/2, ground state,^70^ consistent with the EPR signal of the ammonia-treated S_2_ state. To determine which ammonia binding modes are most consistent with the XAS pre-edge region, we calculated the Mn XAS spectra of the 14  models shown in Figure 4.

**Figure 5:**
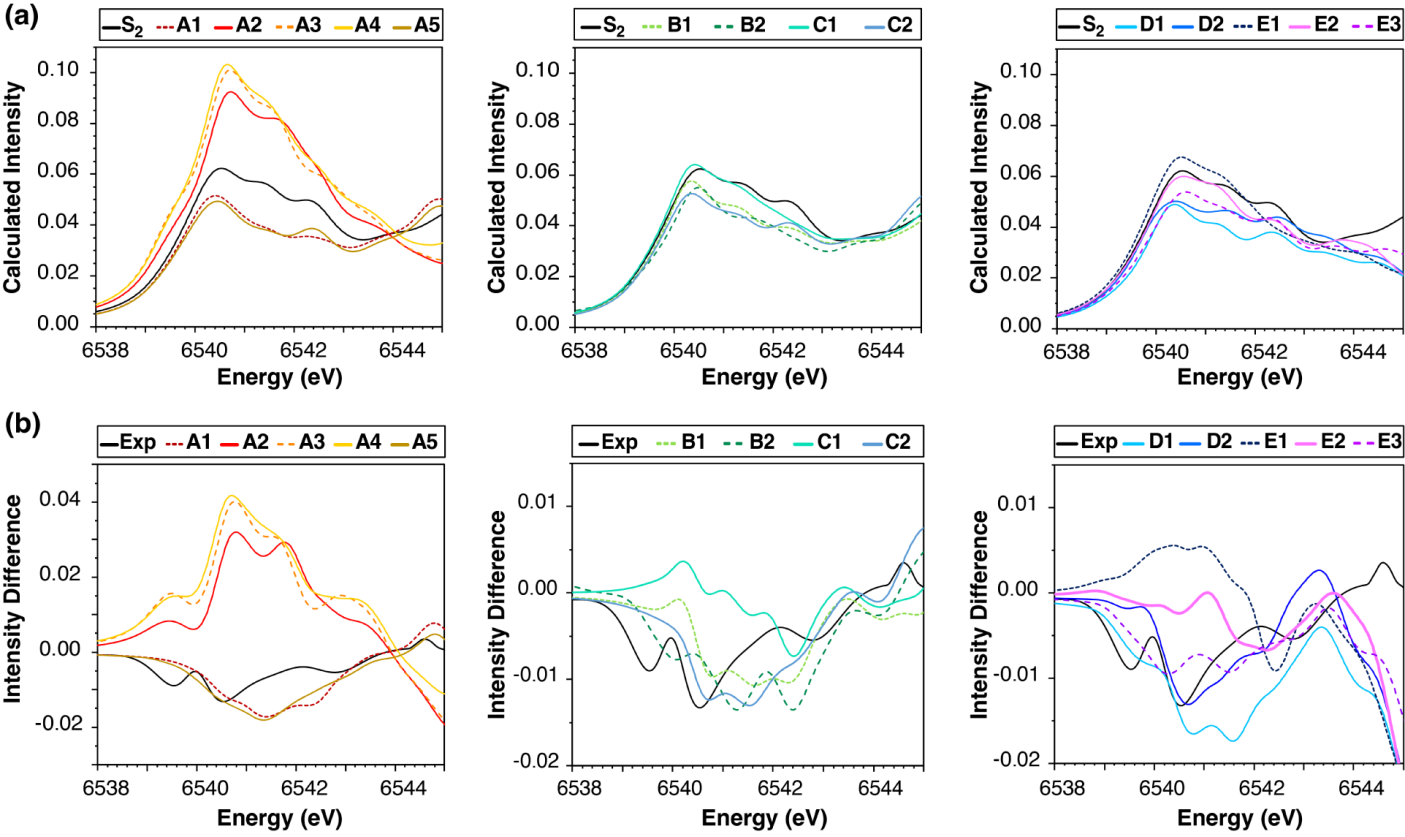
(a) Calculated Mn XAS spectra of the S_2_ state model without ammonia (black) compared to the ammonia-bound S_2_ state models separated in groups A (left), B and C (middle), and D and E (right). (b) Experimental (black) and calculated difference Mn XAS spectra of the ammonia-treated S_2_ state from the untreated S_2_ state, i.e., .

The calculated Mn XAS of the  models compared to the untreated S_2_ state are shown in Figure 5a. To facilitate visualization, models of type A are plotted on the left panel, types B and C in the middle, and types D and E on the right panel. Figure 5a shows the spectra of the S_2_ state model with W1 = W2 = H_2_O protonation states, in line with recent Mn XAS results,^77^ suggesting that both S_1_ and S_2_ feature protonated terminal water ligands. The calculated spectra of the models compared to the S_2_ model with W2 = OH are shown in Figure S6.

Figure 5a shows that all ammonia binding modes include variants that exhibit absorption intensities lower than the S_2_ state, in line with experiment. Nevertheless, several variants can be confidently excluded. Starting with binding mode A, models **A2**, **A3**, and **A4**, where ammonia binds in the O5 position in its deprotonated nitrido form, can be ruled out because their absorption intensity in the 6540–6543 eV region is significantly larger than the S_2_ state. By contrast, ammonia binding in the O5 position in the amido form in models **A1** and **A5** diminishes the absorption intensity. Among the types B–E, models **C1**, **E1**, and **E2** can also be ruled out because the calculated absorption intensities are almost identical to the S_2_ state spectra in the pre-edge region. A more refined comparative analysis for distinguishing between the models based on their similarity to the experimental spectra involves examining the difference spectra of the ammonia-treated samples relative to the untreated S_2_ state, . The difference spectra, shown in Figure 5b, contain exclusively the features that change upon ammonia binding to the OEC.

**Figure 6:**
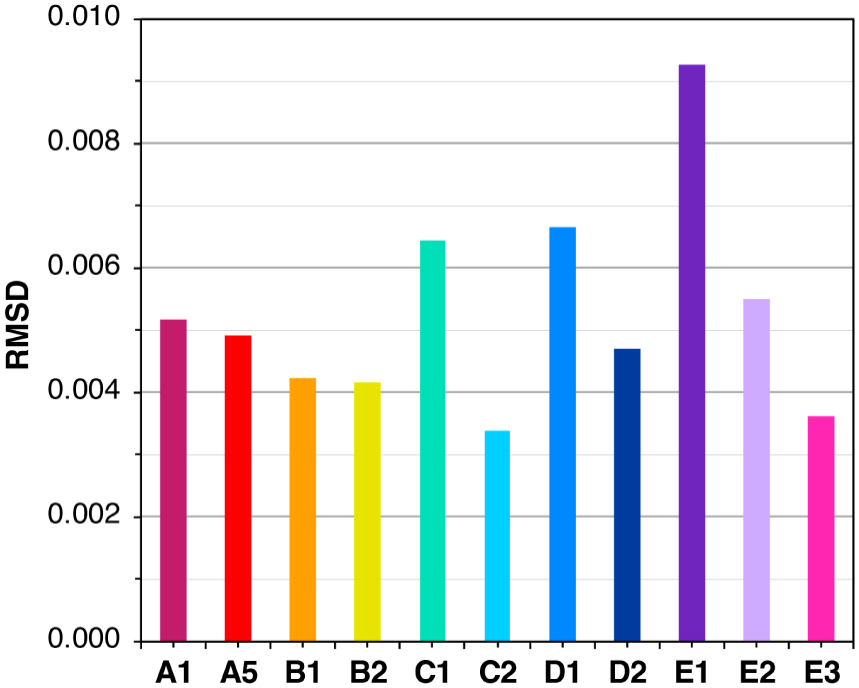
Comparison of RMSDs of the calculated from the experimental  difference spectra for the  models. The RMSD values of **A2**, **A3**, and **A4** are larger than 0.02, and therefore not shown in this figure.

For each  model, the deviation of the calculated  difference spectra from the experimental difference spectra was quantified based on the root mean squared deviation (RMSD) in the region between 6538 and 6544.5 eV. The RMSDs of the  variants are compared in Figure 6, and the numerical values are given in Table S2. The model with the smallest RMSD of 3.38 × 10^–3^ is **C2**, in which ammonia substitutes the terminal W2 ligand on Mn4. Models **B2** and **B1**, in which ammonia substitutes the terminal W1 ligand on Mn4, follow closely, with quite larger RMSD values of 4.15 × 10^–3^ and 4.22 × 10^–3^, respectively. Interestingly, model **E3**, in which ammonia binds to Mn1 and W1 = W2 = H_2_O, also has one of the lowest RMSDs, 3.62 × 10^–3^. Models **E1**, **E2**, **D1**, and **C1** have the largest RMSDs among all  models shown in Figure 6.

Model **C2** was previously found to be the most consistent based on energetic, magnetic, kinetic, and spectroscopic data, while **B1** and **B2** were also close enough to be considered good candidates in terms of these properties.^70^ Therefore, by analyzing the pre-edge region of Mn XAS using quantum chemistry, we arrive at practically the same conclusions as with magnetic spectroscopy data with respect to the ordering of models in terms of their “goodness of fit”. The fact that fundamentally different methods lead to the same conclusions is an important additional argument in favor of the ammonia substitution of a terminal water ligand on Mn4, most probably W2 (model **C2**) or W1 (models **B1**, **B2**).

### 3.3 Electronic Structure Analysis

The data from the TD-DFT calculations enable us to examine in detail the electronic structure differences between the  and S_2_ models that lead to changes in the absorption intensity upon ammonia binding. The XAS spectral shape of each model is a combination of the spectra of all individual Mn sites of the cluster. The XAS spectra of individual Mn ions are plotted in Figure S7. The XAS intensities of each Mn ion correlate strongly with the geometry of its coordination sphere.

**Figure 7:**
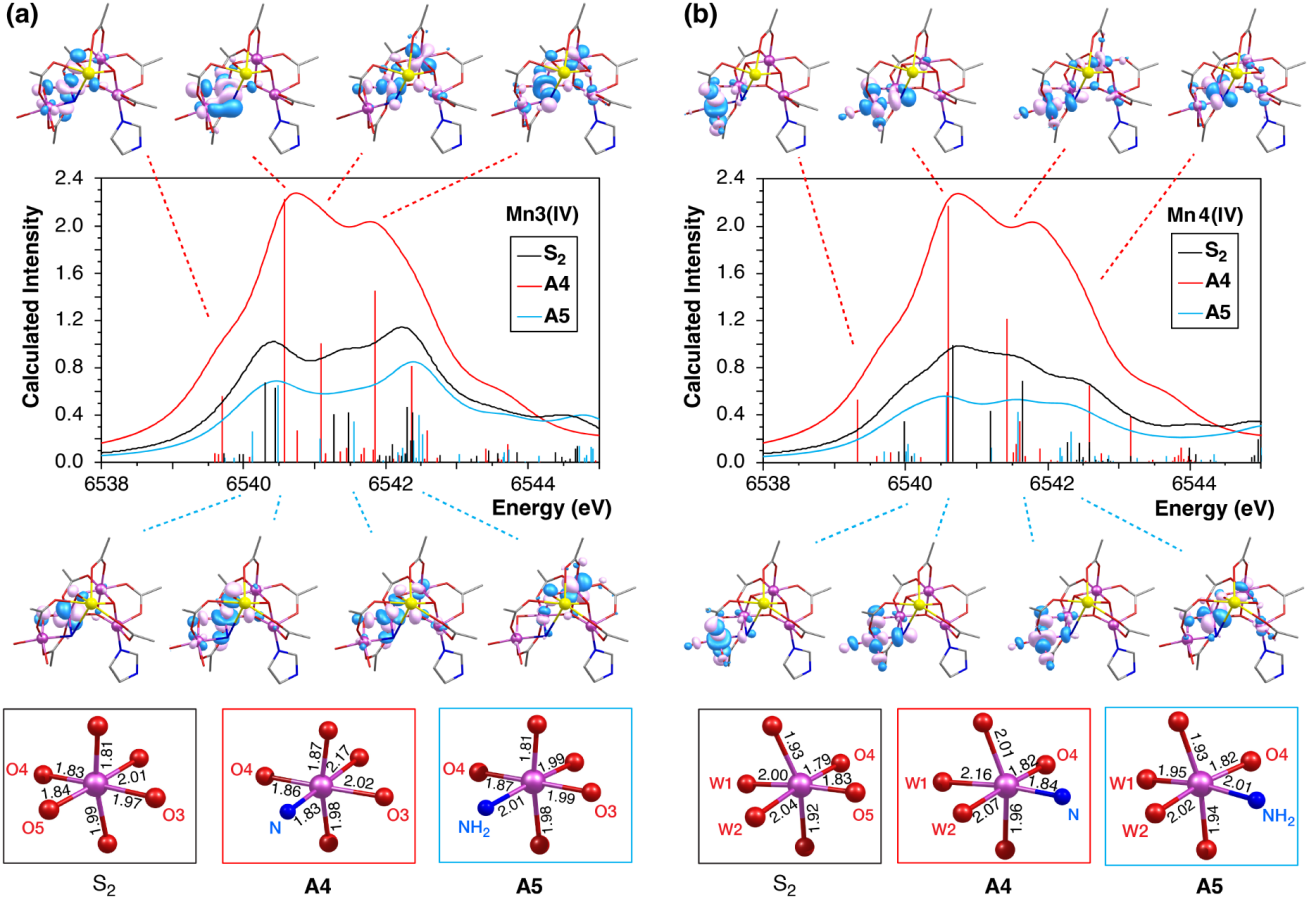
Calculated Mn K-edge XAS spectra: (a) of Mn3 and (b) Mn4 ions of models **A4** (red), **A5** (blue), and of the native S_2_ state (black). The energies and intensities of individual transitions are indicated by colored vertical lines. The acceptor NTOs of the most intense pre-edge transitions of models **A4** and **A5** are also depicted. At the bottom, the coordination sphere and Mn-ligand bond lengths (in Å) of Mn3 (a) and Mn4 (b) ions of each model are shown.

The dramatically higher absorption intensities of the nitrido-substituted models **A2**, **A3**, and **A4** relative to the amido-substituted **A1** and **A5** are exclusively attributed to the Mn3 and Mn4 ions. The stick spectra of the Mn3(IV) and Mn4(IV) ions of models **A4** and **A5** and of the native S_2_ state are compared in Figure 7. The vertical stick heights correspond to the intensities of the transitions. In Figure 7, the natural transition orbitals (NTOs) of the most intense peaks in the region between 6538 and 6542 eV of both Mn3 and Mn4 are plotted. Visual inspection of the NTOs shows that these transitions have predominantly local 1s → 3d character with small contributions from metal-to-metal and metal-to-ligand charge transfer excitations. By contrast, the peaks above 6542 eV are predominantly assigned to metal-to-metal charge transfer excitations. The intensity differences between **A4**, **A5**, and the native S_2_ in the 6539–6542 eV region can be attributed to differences in the local Mn3 and Mn4 geometries and to the nature of the nitrido and amido ligands. The local geometries of the Mn3 and Mn4 ions of **A4**, **A5**, and of the S_2_ state model are also shown in Figure 7. In **A4**, both Mn3 and Mn4 exhibit a pronounced distortion of their octahedral coordination spheres compared to **A5** and S_2_ models. These distortions can be attributed to the strong trans effect of the nitrido (N^3–^) ligand that weakens the opposite Mn3–O and Mn4–O bonds, leading to significant elongation of about 0.2 Å. This deviation from the octahedral geometry allows the mixing of some Mn 4p character into the 3d orbitals, increasing the intensity of the pre-edge region. In addition, a visual comparison of the NTOs of **A4** to those of **A5** reveals that the pre-edge transitions of **A4** have a more pronounced metal-to-ligand charge transfer character with the nitrido ligand playing a more significant role in the excitations than the amido ligand.

**Figure 8:**
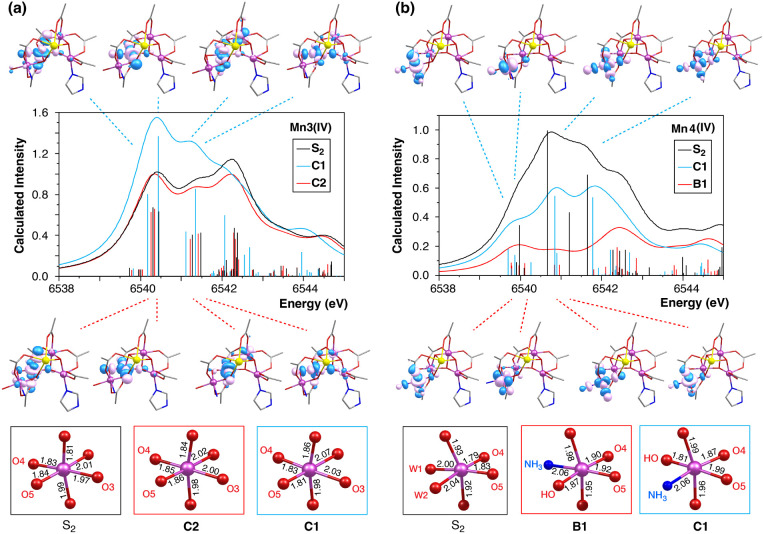
Calculated XAS spectra: (a) of Mn3 ions of models **C1** (blue), **C2** (red), and of the native S_2_ state (black), and (b) of Mn4 ions of models **C1** (blue), **B1** (red), and of the native S_2_ state (black). The energies and intensities of individual transitions are indicated by colored vertical lines. The acceptor NTOs of the most intense pre-edge transitions of models **C1** and **C2** are also depicted. At the bottom, the coordination sphere and Mn-ligand bond lengths (in Å) of the corresponding Mn ions of each model are shown.

The pre-edge intensity differences between the models in which ammonia replaces a terminal water ligand on Mn4, i.e., models of type B and C, correlate to the degrees of distortion from the octahedral geometry around the Mn3 and Mn4 ions. Figure 8 analyzes two characteristic examples. In models **C2** and **C1**, where ammonia substitutes the terminal ligand W2 on Mn4, differences in the protonation state of the W1 terminal ligand lead to variations primarily in the absorption intensities originating from the Mn3 ions. As shown in Figure 8a, the more intense Mn3 1s → 3d transitions in **C1** correlate with a greater distortion in the Mn3 coordination geometry, particularly along the Mn3–O5 axis. Similarly, **C1** shows a higher pre-edge intensity than **B1**, where ammonia binds to Mn4 in the W2 position rather than W1. Here, the origin of this difference lies in the increased degree of distortion around Mn4.

Overall, these results underscore the high sensitivity of XAS to subtle perturbations in the local Mn coordination geometry. This enables distinguishing even between species with the same valence distributions. It is important to note that these structural perturbations result from different types of ammonia binding and the calculated spectra should be insensitive to minor structural perturbations arising from the chosen geometry optimization method. The present results strongly favor ammonia binding modes of type B, C, and E, as well as the possible variants among each mode. Nevertheless, it is important to note that the present analysis cannot explicitly account for the possibility of heterogeneity in the ammonia-treated samples. Specifically, it has been previously suggested based on interpretation of time-resolved detection of O_2_ formation, recombination fluorescence and FTIR experiments,^65^ that two different ammonia-bound S_2_ state forms might coexist in comparable amounts. Based on spectroscopic, magnetic, kinetic, and energetic results these forms were suggested to correspond to terminally substituted W1 and W2 populations.^65,70,108^ As we showed in a recent study,^70^ these models are not easily distinguishable by EPR. The present HERFD XAS results are consistent with both of these binding modes, but suggest that these models are not practically distinguishable by their pre-edge features either. In addition, XAS is consistent with ammonia binding as a sixth ligand on Mn1. However, the latter binding mode was found inconsistent with magnetic spectroscopy data.^70^ Overall, the available experimental data point toward terminal W1/W2 ligand substitution by ammonia and the question of heterogeneity in W1/W2 substitution by ammonia remains open.

## 4 Conclusions

We presented Mn K-edge HERFD XAS of ammonia-bound S_2_ state samples of PSII from *T. vestitus*, used in combination with quantum chemical calculations to distinguish models for ammonia binding to the S_2_ state of the OEC. Mn K-edge XAS spectra were calculated for models representing all proposed ammonia binding modes and compared with experimental difference spectra between ammonia-treated and untreated PSII samples. Our results reveal significant deviations from the experimental spectra in models where ammonia substitutes the bridging μ-oxo ligand O5, especially in its nitrido form. Likewise, the addition of ammonia as a sixth ligand on Mn4 is also found less favorable. The binding mode in which ammonia coordinates as a terminal ligand on Mn4, replacing W2, aligns most closely with the experimental data, followed by models where ammonia replaces the W1 terminal ligand on Mn4. Interestingly, a variant featuring ammonia as an additional sixth ligand on Mn1 also shows one of the lowest deviations from the experimental data. Quantum chemical analysis reveals that the spectral differences among the models originate from the degree of distortion in the Mn octahedral geometry, which gives rise to 4p mixing into Mn 3d orbitals. This structural sensitivity highlights the effectiveness of Mn K-edge HERFD XAS in probing the geometric structure of the OEC, even across variants of the same state and valence distribution. The geometry of the cluster is affected by both the ammonia binding mode and the protonation states of the terminal ligands on Mn4, yielding several possible structural variants. Although the present results do not allow a definitive distinction among the W1 and W2 terminal ligand substitution by ammonia, they offer strong additional and independent support from HERFD XAS for ammonia substitution of a terminal water ligand on Mn4.

## Data Availability

Original data supporting this article are available at Edmond, the Open Research Data Repository of the Max Planck Society at https://doi.org/10.17617/3.8LO0FP.

## Supporting Information Available

Raw experimental data of the XANES and pre-edge regions of ammonia and native S_1_ and S_2_ (Figure S1); comparison of the raw and binned spectra of the pre-edge (Figure S2); fitting of the pre-edge with Voigt curves (Figure S3); comparison of the raw, binned and fitted XAS spectra (Figure S4); experimental and calculated S_2_ – S_1_ difference spectra (Figure S5); parameters of Voigt curves used for fitting the pre-edge (Table S1); calculated XAS spectra and difference spectra compared to the native S_2_ state model with W2 = OH (Figure S6); calculated XAS spectra for individual Mn ions (Figure S7); RMSDs of the difference spectra (Table S2); Orca sample input file for XAS calculations (PDF)

## References

[ref1] Shevela D.; Bjorn L. O. Photosynthesis: Solar Energy for Life; World Scientific Publishing: Singapore, 2018; p 204.

[ref2] Blankenship R. E. Molecular mechanisms of photosynthesis, 3rd Ed.; John Wiley & Sons: Chichester, 2021; p 352.

[ref3] Shevela D.; Kern J. F.; Govindjee G.; Messinger J. Solar energy conversion by photosystem II: principles and structures. Photosynth. Res. 2023, 156, 279–307. 10.1007/s11120-022-00991-y. 36826741 PMC10203033

[ref4] Pantazis D. A. In Solar-to-Chemical Conversion: Photocatalytic and Photoelectrochemical Processes; Sun H., Ed.; Wiley: 2021, pp. 41–76.

[ref5] Lubitz W.; Chrysina M.; Cox N. Water oxidation in photosystem II. Photosynth. Res. 2019, 142, 105–125. 10.1007/s11120-019-00648-3. 31187340 PMC6763417

[ref6] Yano J.; Yachandra V. Mn_4_Ca Cluster in Photosynthesis: Where and How Water is Oxidized to Dioxygen. Chem. Rev 2014, 114, 4175–4205. 10.1021/cr4004874. 24684576 PMC4002066

[ref7] Shen J.-R. The Structure of Photosystem II and the Mechanism of Water Oxidation in Photosynthesis. Annu. Rev. Plant Biol. 2015, 66, 23–48. 10.1146/annurev-arplant-050312-120129. 25746448

[ref8] Joliot P.; Barbieri G.; Chabaud R. Un nouveau modele des centres photochimiques du systeme II. Photochem. Photobiol. 1969, 10, 309–329. 10.1111/j.1751-1097.1969.tb05696.x.

[ref9] Kok B.; Forbush B.; McGloin M. Cooperation of charges in photosynthetic O_2_ evolution. Photochem. Photobiol. 1970, 11, 457–475. 10.1111/j.1751-1097.1970.tb06017.x. 5456273

[ref10] Suzuki H.; Sugiura M.; Noguchi T. Monitoring Water Reactions during the S-State Cycle of the Photosynthetic Water-Oxidizing Center: Detection of the DOD Bending Vibrations by Means of Fourier Transform Infrared Spectroscopy. Biochemistry 2008, 47, 11024–11030. 10.1021/bi801580e. 18821774

[ref11] Nilsson H.; Krupnik T.; Kargul J.; Messinger J. Substrate water exchange in photosystem II core complexes of the extremophilic red alga *Cyanidioschyzon merolae*. Biochim. Biophys. Acta, Bioenerg. 2014, 1837, 1257–1262. 10.1016/j.bbabio.2014.04.001. 24726350

[ref12] Isobe H.; Shoji M.; Shen J.-R.; Yamaguchi K. Strong Coupling between the Hydrogen Bonding Environment and Redox Chemistry during the S_2_ to S_3_ Transition in the Oxygen-Evolving Complex of Photosystem II. J. Phys. Chem. B 2015, 119, 13922–13933. 10.1021/acs.jpcb.5b05740. 26440915

[ref13] Shoji M.; Isobe H.; Yamaguchi K. QM/MM study of the S_2_ to S_3_ transition reaction in the oxygen-evolving complex of photosystem II. Chem. Phys. Lett. 2015, 636, 172–179. 10.1016/j.cplett.2015.07.039. 26440915

[ref14] Retegan M.; Krewald V.; Mamedov F.; Neese F.; Lubitz W.; Cox N.; Pantazis D. A. A five-coordinate Mn(IV) intermediate in biological water oxidation: spectroscopic signature and a pivot mechanism for water binding. Chem. Sci 2016, 7, 72–84. 10.1039/C5SC03124A. 29861966 PMC5950799

[ref15] Capone M.; Narzi D.; Bovi D.; Guidoni L. Mechanism of Water Delivery to the Active Site of Photosystem II along the S_2_ to S_3_ Transition. J. Phys. Chem. Lett. 2016, 7, 592–596. 10.1021/acs.jpclett.5b02851. 26799278

[ref16] Sakamoto H.; Shimizu T.; Nagao R.; Noguchi T. Monitoring the Reaction Process During the S_2_ → S_3_ Transition in Photosynthetic Water Oxidation Using Time-Resolved Infrared Spectroscopy. J. Am. Chem. Soc. 2017, 139, 2022–2029. 10.1021/jacs.6b11989. 28088851

[ref17] de Lichtenberg C.; Messinger J. Substrate water exchange in the S_2_ state of photosystem II is dependent on the conformation of the Mn_4_Ca cluster. Phys. Chem. Chem. Phys. 2020, 22, 12894–12908. 10.1039/D0CP01380C. 32373850

[ref18] de Lichtenberg C.; Kim C. J.; Chernev P.; Debus R. J.; Messinger J. The exchange of the fast substrate water in the S_2_ state of photosystem II is limited by diffusion of bulk water through channels – implications for the water oxidation mechanism. Chem. Sci. 2021, 12, 12763–12775. 10.1039/D1SC02265B. 34703563 PMC8494045

[ref19] Wang J.; Askerka M.; Brudvig G. W.; Batista V. S. Crystallographic Data Support the Carousel Mechanism of Water Supply to the Oxygen-Evolving Complex of Photosystem II. ACS Energy Lett. 2017, 2, 2299–2306. 10.1021/acsenergylett.7b00750. 29057331 PMC5644713

[ref20] Pantazis D. A. The S_3_ State of the Oxygen-Evolving Complex: Overview of Spectroscopy and XFEL Crystallography with a Critical Evaluation of Early-Onset Models for O–O Bond Formation. Inorganics 2019, 7, 55 10.3390/inorganics7040055.

[ref21] Liu J.; Yang K. R.; Long Z.; Armstrong W. H.; Brudvig G. W.; Batista V. S. Water Ligands Regulate the Redox Leveling Mechanism of the Oxygen-Evolving Complex of the Photosystem II. J. Am. Chem. Soc. 2024, 146, 15986–15999. 10.1021/jacs.4c02926. 38833517

[ref22] Siegbahn P. E. M. Water oxidation mechanism in photosystem II, including oxidations, proton release pathways, O–O bond formation and O_2_ release. Biochim. Biophys. Acta, Bioenerg. 2013, 1827, 1003–1019. 10.1016/j.bbabio.2012.10.006. 23103385

[ref23] Shoji M.; Isobe H.; Shigeta Y.; Nakajima T.; Yamaguchi K. Nonadiabatic one-electron transfer mechanism for the O–O bond formation in the oxygen-evolving complex of photosystem II. Chem. Phys. Lett. 2018, 698, 138–146. 10.1016/j.cplett.2018.02.056.

[ref24] Capone M.; Narzi D.; Guidoni L. Mechanism of Oxygen Evolution and Mn_4_CaO_5_ Cluster Restoration in the Natural Water-Oxidizing Catalyst. Biochemistry 2021, 60, 2341–2348. 10.1021/acs.biochem.1c00226. 34283569

[ref25] Simon P. S.; Makita H.; Bogacz I.; Fuller F.; Bhowmick A.; Hussein R.; Ibrahim M.; Zhang M.; Chatterjee R.; Cheah M. H.; et al. Capturing the sequence of events during the water oxidation reaction in photosynthesis using XFELs. FEBS Lett. 2023, 597, 30–37. 10.1002/1873-3468.14527. 36310373 PMC9839502

[ref26] Greife P.; Schonborn M.; Capone M.; Assuncao R.; Narzi D.; Guidoni L.; Dau H. The electron-proton bottleneck of photosynthetic oxygen evolution. Nature 2023, 617, 623–628. 10.1038/s41586-023-06008-5. 37138082 PMC10191853

[ref27] Guo Y.; Messinger J.; Kloo L.; Sun L. Alternative Mechanism for O_2_ Formation in Natural Photosynthesis via Nucleophilic Oxo–Oxo Coupling. J. Am. Chem. Soc. 2023, 145, 4129–4141. 10.1021/jacs.2c12174. 36763485

[ref28] Capone M.; Guidoni L.; Narzi D. Structural and dynamical characterization of the S_4_ state of the Kok-Joliot’s cycle by means of QM/MM Molecular Dynamics Simulations. Chem. Phys. Lett. 2020, 742, 137111 10.1016/j.cplett.2020.137111.

[ref29] Guo Y.; He L.; Ding Y.; Kloo L.; Pantazis D. A.; Messinger J.; Sun L. Closing Kok’s cycle of nature’s water oxidation catalysis. Nat. Commun. 2024, 15, 5982 10.1038/s41467-024-50210-6. 39013902 PMC11252165

[ref30] Murray J. W.; Barber J. Structural characteristics of channels and pathways in photosystem II including the identification of an oxygen channel. J. Struct. Biol. 2007, 159, 228–237. 10.1016/j.jsb.2007.01.016. 17369049

[ref31] Ho F. M.; Styring S. Access channels and methanol binding site to the CaMn_4_ cluster in Photosystem II based on solvent accessibility simulations, with implications for substrate water access. Biochim. Biophys. Acta, Bioenerg. 2008, 1777, 140–153. 10.1016/j.bbabio.2007.08.009. 17964532

[ref32] Gabdulkhakov A.; Guskov A.; Broser M.; Kern J.; Müh F.; Saenger W.; Zouni A. Probing the Accessibility of the Mn_4_Ca Cluster in Photosystem II: Channels Calculation, Noble Gas Derivatization, and Cocrystallization with DMSO. Structure 2009, 17, 1223–1234. 10.1016/j.str.2009.07.010. 19748343

[ref33] Vassiliev S.; Comte P.; Mahboob A.; Bruce D. Tracking the Flow of Water through Photosystem II Using Molecular Dynamics and Streamline Tracing. Biochemistry 2010, 49, 1873–1881. 10.1021/bi901900s. 20121111

[ref34] Ho F. M. In Molecular solar fuels; Wydrzynski T. J.; Hillier W., Eds.; RCS Publishing: Cambridge, 2012; pp. 208–248.

[ref35] Vassiliev S.; Zaraiskaya T.; Bruce D. Exploring the energetics of water permeation in photosystem II by multiple steered molecular dynamics simulations. Biochim. Biophys. Acta, Bioenerg. 2012, 1817, 1671–1678. 10.1016/j.bbabio.2012.05.016. 22683291

[ref36] Kaur D.; Zhang Y.; Reiss K. M.; Mandal M.; Brudvig G. W.; Batista V. S.; Gunner M. R. Proton exit pathways surrounding the oxygen evolving complex of photosystem II. Biochim. Biophys. Acta, Bioenerg. 2021, 1862, 148446 10.1016/j.bbabio.2021.148446. 33964279

[ref37] Sirohiwal A.; Pantazis D. A. Functional Water Networks in Fully Hydrated Photosystem II. J. Am. Chem. Soc. 2022, 144, 22035–22050. 10.1021/jacs.2c09121. 36413491 PMC9732884

[ref38] Doyle M. D.; Bhowmick A.; Wych D. C.; Lassalle L.; Simon P. S.; Holton J.; Sauter N. K.; Yachandra V. K.; Kern J. F.; Yano J.; et al. Water Networks in Photosystem II Using Crystalline Molecular Dynamics Simulations and Room-Temperature XFEL Serial Crystallography. J. Am. Chem. Soc. 2023, 145, 14621–14635. 10.1021/jacs.3c01412. 37369071 PMC10347547

[ref39] Hussein R.; Graça A.; Forsman J.; Aydin A. O.; Hall M.; Gaetcke J.; Chernev P.; Wendler P.; Dobbek H.; Messinger J.; et al. Cryo–electron microscopy reveals hydrogen positions and water networks in photosystem II. Science 2024, 384, 1349–1355. 10.1126/science.adn6541. 38900892

[ref40] Cox N.; Messinger J. Reflections on substrate water and dioxygen formation. Biochim. Biophys. Acta, Bioenerg. 2013, 1827, 1020–1030. 10.1016/j.bbabio.2013.01.013. 23380392

[ref41] Pantazis D. A. Missing Pieces in the Puzzle of Biological Water Oxidation. ACS Catal. 2018, 8, 9477–9507. 10.1021/acscatal.8b01928.

[ref42] Cox N.; Pantazis D. A.; Lubitz W. Current Understanding of the Mechanism of Water Oxidation in Photosystem II and Its Relation to XFEL Data. Annu. Rev. Biochem. 2020, 89, 795–820. 10.1146/annurev-biochem-011520-104801. 32208765

[ref43] Hussein R.; Ibrahim M.; Bhowmick A.; Simon P. S.; Bogacz I.; Doyle M. D.; Dobbek H.; Zouni A.; Messinger J.; Yachandra V. K.; et al. Evolutionary diversity of proton and water channels on the oxidizing side of photosystem II and their relevance to function. Photosynth. Res. 2023, 158, 91–107. 10.1007/s11120-023-01018-w. 37266800 PMC10684718

[ref44] Force D. A.; Randall D. W.; Lorigan G. A.; Clemens K. L.; Britt R. D. ESEEM Studies of Alcohol Binding to the Manganese Cluster of the Oxygen Evolving Complex of Photosystem II. J. Am. Chem. Soc. 1998, 120, 13321–13333. 10.1021/ja982713b.

[ref45] Åhrling K. A.; Evans M. C. W.; Nugent J. H. A.; Ball R. J.; Pace R. J. ESEEM Studies of Substrate Water and Small Alcohol Binding to the Oxygen-Evolving Complex of Photosystem II during Functional Turnover. Biochemistry 2006, 45, 7069–7082. 10.1021/bi052146m. 16752897

[ref46] Nöring B.; Shevela D.; Renger G.; Messinger J. Effects of methanol on the S*_i_*-state transitions in photosynthetic water-splitting. Photosynth. Res. 2008, 98, 251–260. 10.1007/s11120-008-9364-4. 18819015

[ref47] Perez Navarro M.; Ames W. M.; Nilsson H.; Lohmiller T.; Pantazis D. A.; Rapatskiy L.; Nowaczyk M. M.; Neese F.; Boussac A.; Messinger J.; et al. Ammonia binding to the oxygen-evolving complex of photosystem II identifies the solvent-exchangeable oxygen bridge (*μ*-oxo) of the manganese tetramer. Proc. Natl. Acad. Sci. U. S. A. 2013, 110, 15561–15566. 10.1073/pnas.1304334110. 24023065 PMC3785721

[ref48] Oyala P. H.; Stich T. A.; Stull J. A.; Yu F.; Pecoraro V. L.; Britt R. D. Pulse Electron Paramagnetic Resonance Studies of the Interaction of Methanol with the S_2_ State of the Mn_4_O_5_Ca Cluster of Photosystem II. Biochemistry 2014, 53, 7914–7928. 10.1021/bi501323h. 25441091 PMC5689085

[ref49] Lohmiller T.; Krewald V.; Navarro M. P.; Retegan M.; Rapatskiy L.; Nowaczyk M. M.; Boussac A.; Neese F.; Lubitz W.; Pantazis D. A.; et al. Structure, ligands and substrate coordination of the oxygen-evolving complex of photosystem II in the S_2_ state: a combined EPR and DFT study. Phys. Chem. Chem. Phys. 2014, 16, 11877–11892. 10.1039/c3cp55017f. 24525937

[ref50] Oyala P. H.; Stich T. A.; Debus R. J.; Britt R. D. Ammonia Binds to the Dangler Manganese of the Photosystem II Oxygen-Evolving Complex. J. Am. Chem. Soc. 2015, 137, 8829–8837. 10.1021/jacs.5b04768. 26083545

[ref51] Retegan M.; Pantazis D. A. Interaction of methanol with the oxygen-evolving complex: atomistic models, channel identification, species dependence, and mechanistic implications. Chem. Sci. 2016, 7, 6463–6476. 10.1039/C6SC02340A. 28451104 PMC5355959

[ref52] Askerka M.; Brudvig G. W.; Batista V. S. The O_2_ -Evolving Complex of Photosystem II: Recent Insights from Quantum Mechanics/Molecular Mechanics (QM/MM), Extended X-ray Absorption Fine Structure (EXAFS), and Femtosecond X-ray Crystallography Data. Acc. Chem. Res. 2017, 50, 41–48. 10.1021/acs.accounts.6b00405. 28001034

[ref53] Nagashima H.; Mino H. Location of Methanol on the S_2_ State Mn Cluster in Photosystem II Studied by Proton Matrix Electron Nuclear Double Resonance. J. Phys. Chem. Lett. 2017, 8, 621–625. 10.1021/acs.jpclett.7b00110. 28099021

[ref54] Retegan M.; Pantazis D. A. Differences in the Active Site of Water Oxidation among Photosynthetic Organisms. J. Am. Chem. Soc. 2017, 139, 14340–14343. 10.1021/jacs.7b06351. 28948784

[ref55] Marchiori D. A.; Oyala P. H.; Debus R. J.; Stich T. A.; Britt R. D. Structural Effects of Ammonia Binding to the Mn_4_CaO_5_ Cluster of Photosystem II. J. Phys. Chem. B 2018, 122, 1588–1599. 10.1021/acs.jpcb.7b11101. 29303579

[ref56] Sandusky P. O.; Yocum C. F. The chloride requirement for photosynthetic oxygen evolution. Analysis of the effects of chloride and other anions on amine inhibition of the oxygen-evolving complex. Biochim. Biophys. Acta, Bioenerg. 1984, 766, 603–611. 10.1016/0005-2728(84)90121-X.

[ref57] Sandusky P. O.; Yocum C. F. The chloride requirement for photosynthetic oxygen evolution: Factors affecting nucleophilic displacement of chloride from the oxygen-evolving complex. Biochim. Biophys. Acta, Bioenerg. 1986, 849, 85–93. 10.1016/0005-2728(86)90099-X.

[ref58] Vinyard D. J.; Askerka M.; Debus R. J.; Batista V. S.; Brudvig G. W. Ammonia Binding in the Second Coordination Sphere of the Oxygen-Evolving Complex of Photosystem II. Biochemistry 2016, 55, 4432–4436. 10.1021/acs.biochem.6b00543. 27433995

[ref59] Mandal M.; Askerka M.; Banerjee G.; Amin M.; Brudvig G. W.; Batista V. S.; Gunner M. R. Characterization of ammonia binding to the second coordination shell of the oxygen-evolving complex of photosystem II. Dalton Trans. 2017, 46, 16089–16095. 10.1039/C7DT03901H. 29120469

[ref60] Beck W. F.; De Paula J. C.; Brudvig G. W. Ammonia binds to the manganese site of the oxygen-evolving complex of photosystem II in the S_2_ state. J. Am. Chem. Soc. 1986, 108, 4018–4022. 10.1021/ja00274a027.

[ref61] Britt R. D.; Zimmermann J. L.; Sauer K.; Klein M. P. Ammonia binds to the catalytic manganese of the oxygen-evolving complex of photosystem II. Evidence by electron spin-echo envelope modulation spectroscopy. J. Am. Chem. Soc. 1989, 111, 3522–3532. 10.1021/ja00192a006.

[ref62] Sandusky P. O.; Yocum C. F. The mechanism of amine inhibition of the photosynthetic oxygen evolving complex: Amines displace functional chloride from a ligand site on manganese. FEBS Lett. 1983, 162, 339–343. 10.1016/0014-5793(83)80784-4.

[ref63] Boussac A.; Rutherford A. W.; Styring S. Interaction of ammonia with the water splitting enzyme of photosystem II. Biochemistry 1990, 29, 24–32. 10.1021/bi00453a003. 2157480

[ref64] Zimmermann J. L.; Rutherford A. W. Electron paramagnetic resonance properties of the S_2_ state of the oxygen-evolving complex of photosystem II. Biochemistry 1986, 25, 4609–4615. 10.1021/bi00364a023.

[ref65] Schuth N.; Liang Z.; Schönborn M.; Kussicke A.; Assunção R.; Zaharieva I.; Zilliges Y.; Dau H. Inhibitory and Non-Inhibitory NH_3_ Binding at the Water-Oxidizing Manganese Complex of Photosystem II Suggests Possible Sites and a Rearrangement Mode of Substrate Water Molecules. Biochemistry 2017, 56, 6240–6256. 10.1021/acs.biochem.7b00743. 29086556

[ref66] Hou L.-H.; Wu C.-M.; Huang H.-H.; Chu H.-A. Effects of Ammonia on the Structure of the Oxygen-Evolving Complex in Photosystem II As Revealed by Light-Induced FTIR Difference Spectroscopy. Biochemistry 2011, 50, 9248–9254. 10.1021/bi200943q. 21942297

[ref67] Vinyard D. J.; Brudvig G. W. Insights into Substrate Binding to the Oxygen-Evolving Complex of Photosystem II from Ammonia Inhibition Studies. Biochemistry 2015, 54, 622–628. 10.1021/bi5014134. 25531753

[ref68] Pokhrel R.; Brudvig G. W. Oxygen-evolving complex of photosystem II: correlating structure with spectroscopy. Phys. Chem. Chem. Phys. 2014, 16, 11812–11821. 10.1039/c4cp00493k. 24700294

[ref69] Guo Y.; He L.-L.; Zhao D.-X.; Gong L.-D.; Liu C.; Yang Z.-Z. How does ammonia bind to the oxygen-evolving complex in the S_2_ state of photosynthetic water oxidation? Theoretical support and implications for the W1 substitution mechanism. Phys. Chem. Chem. Phys. 2016, 18, 31551–31565. 10.1039/C6CP05725J. 27831574

[ref70] Drosou M.; Pantazis D. A. Comprehensive Evaluation of Models for Ammonia Binding to the Oxygen Evolving Complex of Photosystem II. J. Phys. Chem. B 2024, 128, 1333–1349. 10.1021/acs.jpcb.3c06304. 38299511 PMC10875651

[ref71] Schraut J.; Kaupp M. On Ammonia Binding to the Oxygen-Evolving Complex of Photosystem II: A Quantum Chemical Study. Chem.-Eur. J. 2014, 20, 7300–7308. 10.1002/chem.201304464. 24806267

[ref72] Askerka M.; Vinyard D. J.; Brudvig G. W.; Batista V. S. NH_3_ Binding to the S_2_ State of the O_2_-Evolving Complex of Photosystem II: Analogue to H_2_O Binding during the S_2_ → S_3_ Transition. Biochemistry 2015, 54, 5783–5786. 10.1021/acs.biochem.5b00974. 26378340

[ref73] Pushkar Y.; Ravari A. K.; Jensen S. C.; Palenik M. Early Binding of Substrate Oxygen Is Responsible for a Spectroscopically Distinct S_2_ State in Photosystem II. J. Phys. Chem. Lett. 2019, 10, 5284–5291. 10.1021/acs.jpclett.9b01255. 31419136

[ref74] Young I. D.; Ibrahim M.; Chatterjee R.; Gul S.; Fuller F.; Koroidov S.; Brewster A. S.; Tran R.; Alonso-Mori R.; Kroll T.; et al. Structure of photosystem II and substrate binding at room temperature. Nature 2016, 540, 453–457. 10.1038/nature20161. 27871088 PMC5201176

[ref75] Dau H.; Andrews J. C.; Roelofs T. A.; Latimer M. J.; Liang W.; Yachandra V. K.; Sauer K.; Klein M. P. Structural Consequences of Ammonia Binding to the Manganese Center of the Photosynthetic Oxygen-Evolving Complex: An X-ray Absorption Spectroscopy Study of Isotropic and Oriented Photosystem II Particles. Biochemistry 1995, 34, 5274–5287. 10.1021/bi00015a043. 7711049

[ref76] Mermigki M. A.; Drosou M.; Pantazis D. A. On the nature of high-spin forms in the S_2_ state of the oxygen-evolving complex. Chem. Sci. 2025, 16, 4023–4047. 10.1039/D4SC07818G. 39898302 PMC11784572

[ref77] Chrysina M.; Drosou M.; Castillo R. G.; Reus M.; Neese F.; Krewald V.; Pantazis D. A.; DeBeer S. Nature of S-States in the Oxygen-Evolving Complex Resolved by High-Energy Resolution Fluorescence Detected X-ray Absorption Spectroscopy. J. Am. Chem. Soc. 2023, 145, 25579–25594. 10.1021/jacs.3c06046. 37970825 PMC10690802

[ref78] Messinger J.; Robblee J. H.; Bergmann U.; Fernandez C.; Glatzel P.; Visser H.; Cinco R. M.; McFarlane K. L.; Bellacchio E.; Pizarro S. A.; et al. Absence of Mn-centered oxidation in the S_2_ → S_3_ transition: implications for the mechanism of photosynthetic water oxidation. J. Am. Chem. Soc. 2001, 123, 7804–7820. 10.1021/ja004307+. 11493054 PMC3965774

[ref79] Haumann M.; Muller C.; Liebisch P.; Iuzzolino L.; Dittmer J.; Grabolle M.; Neisius T.; Meyer-Klaucke W.; Dau H. Structural and oxidation state changes of the photosystem II manganese complex in four transitions of the water oxidation cycle (S_0_ → S_1_, S_1_ → S_2_, S_2_ → S_3_, and S_3,4_ → S_0_) characterized by X-ray absorption spectroscopy at 20 K and room temperature. Biochemistry 2005, 44, 1894–1908. 10.1021/bi048697e. 15697215

[ref80] Glatzel P.; Schroeder H.; Pushkar Y.; Boron T. III; Mukherjee S.; Christou G.; Pecoraro V. L.; Messinger J.; Yachandra V. K.; Bergmann U.; et al. Electronic Structural Changes of Mn in the Oxygen-Evolving Complex of Photosystem II during the Catalytic Cycle. Inorg. Chem. 2013, 52, 5642–5644. 10.1021/ic4005938. 23647530 PMC3683399

[ref81] de Groot F. High-Resolution X-ray Emission and X-ray Absorption Spectroscopy. Chem. Rev. 2001, 101, 1779–1808. 10.1021/cr9900681. 11709999

[ref82] Hämäläinen K.; Siddons D. P.; Hastings J. B.; Berman L. E. Elimination of the inner-shell lifetime broadening in X-ray-absorption spectroscopy. Phys. Rev. Lett. 1991, 67, 2850–2853. 10.1103/PhysRevLett.67.2850. 10044570

[ref83] Cutsail G. E. III; DeBeer S. Challenges and Opportunities for Applications of Advanced X-ray Spectroscopy in Catalysis Research. ACS Catal. 2022, 12, 5864–5886. 10.1021/acscatal.2c01016.

[ref84] Glatzel P.; Weng T.-C.; Kvashnina K.; Swarbrick J.; Sikora M.; Gallo E.; Smolentsev N.; Mori R. A. Reflections on hard X-ray photon-in/photon-out spectroscopy for electronic structure studies. J. Electron Spectrosc. Relat. Phenom. 2013, 188, 17–25. 10.1016/j.elspec.2012.09.004.

[ref85] Kuhl H.; Kruip J.; Seidler A.; Krieger-Liszkay A.; Bunker M.; Bald D.; Scheidig A. J.; Rogner M. Towards structural determination of the water-splitting enzyme. Purification, crystallization, and preliminary crystallographic studies of photosystem II from a thermophilic cyanobacterium. J. Biol. Chem. 2000, 275, 20652–20659. 10.1074/jbc.M001321200. 10748017

[ref86] Tanaka A.; Fukushima Y.; Kamiya N. Two Different Structures of the Oxygen-Evolving Complex in the Same Polypeptide Frameworks of Photosystem II. J. Am. Chem. Soc. 2017, 139, 1718–1721. 10.1021/jacs.6b09666. 28102667

[ref87] Neese F.; Wennmohs F.; Becker U.; Riplinger C. The ORCA quantum chemistry program package. J. Chem. Phys. 2020, 152 (22), 224108 10.1063/5.0004608. 32534543

[ref88] DeBeer George S.; Petrenko T.; Neese F. Prediction of Iron K-Edge Absorption Spectra Using Time-Dependent Density Functional Theory. J. Phys. Chem. A 2008, 112, 12936–12943. 10.1021/jp803174m. 18698746

[ref89] Roemelt M.; Beckwith M. A.; Duboc C.; Collomb M.-N.; Neese F.; DeBeer S. Manganese K-Edge X-Ray Absorption Spectroscopy as a Probe of the Metal–Ligand Interactions in Coordination Compounds. Inorg. Chem. 2012, 51, 680–687. 10.1021/ic202229b. 22145735

[ref90] Krewald V.; Neese F.; Pantazis D. A. On the Magnetic and Spectroscopic Properties of High-Valent Mn_3_CaO_4_ Cubanes as Structural Units of Natural and Artificial Water-Oxidizing Catalysts. J. Am. Chem. Soc. 2013, 135, 5726–5739. 10.1021/ja312552f. 23527603

[ref91] Beckwith M. A.; Ames W.; Vila F. D.; Krewald V.; Pantazis D. A.; Mantel C.; Pécaut J.; Gennari M.; Duboc C.; Collomb M.-N.; et al. How Accurately Can Extended X-ray Absorption Spectra Be Predicted from First Principles? Implications for Modeling the Oxygen-Evolving Complex in Photosystem II. J. Am. Chem. Soc. 2015, 137, 12815–12834. 10.1021/jacs.5b00783. 26352328

[ref92] Hirata S.; Head-Gordon M. Time-dependent density functional theory within the Tamm–Dancoff approximation. Chem. Phys. Lett. 1999, 314, 291–299. 10.1016/S0009-2614(99)01149-5.

[ref93] Staroverov V. N.; Scuseria G. E.; Tao J.; Perdew J. P. Comparative assessment of a new nonempirical density functional: Molecules and hydrogen-bonded complexes. J. Chem. Phys. 2003, 119, 12129–12137. 10.1063/1.1626543.

[ref94] van Lenthe E.; Baerends E. J.; Snijders J. G. Relativistic regular two-component Hamiltonians. J. Chem. Phys. 1993, 99, 4597–4610. 10.1063/1.465190.

[ref95] van Lenthe E.; Baerends E. J.; Snijders J. G. Relativistic total energy using regular approximations. J. Chem. Phys. 1994, 101, 9783–9792. 10.1063/1.467943.

[ref96] van Wüllen C. Molecular density functional calculations in the regular relativistic approximation: Method, application to coinage metal diatomics, hydrides, fluorides and chlorides, and comparison with first-order relativistic calculations. J. Chem. Phys. 1998, 109, 392–399. 10.1063/1.476576.

[ref97] van Lenthe E.; Snijders J. G.; Baerends E. J. The zero-order regular approximation for relativistic effects: The effect of spin–orbit coupling in closed shell molecules. J. Chem. Phys. 1996, 105, 6505–6516. 10.1063/1.472460.

[ref98] Pantazis D. A.; Chen X.-Y.; Landis C. R.; Neese F. All-Electron Scalar Relativistic Basis Sets for Third-Row Transition Metal Atoms. J. Chem. Theory Comput. 2008, 4, 908–919. 10.1021/ct800047t. 26621232

[ref99] Weigend F.; Ahlrichs R. Balanced basis sets of split valence, triple zeta valence and quadruple zeta valence quality for H to Rn: Design and assessment of accuracy. Phys. Chem. Chem. Phys. 2005, 7, 3297–3305. 10.1039/b508541a. 16240044

[ref100] Barone V.; Cossi M. Quantum Calculation of Molecular Energies and Energy Gradients in Solution by a Conductor Solvent Model. J. Phys. Chem. A 1998, 102, 1995–2001. 10.1021/jp9716997.

[ref101] Neese F.; Olbrich G. Efficient use of the resolution of the identity approximation in time-dependent density functional calculations with hybrid density functionals. Chem. Phys. Lett. 2002, 362, 170–178. 10.1016/S0009-2614(02)01053-9.

[ref102] Neese F.; Wennmohs F.; Hansen A.; Becker U. Efficient, approximate and parallel Hartree–Fock and hybrid DFT calculations. A ‘chain-of-spheres’ algorithm for the Hartree–Fock exchange. J. Chem. Phys. 2009, 356, 98–109. 10.1016/j.chemphys.2008.10.036.

[ref103] Weigend F. Accurate Coulomb-fitting basis sets for H to Rn. Phys. Chem. Chem. Phys. 2006, 8, 1057–1065. 10.1039/b515623h. 16633586

[ref104] Dismukes G. C.; Siderer Y. EPR spectroscopic observations of a manganese center associated with water oxidation in spinach chloroplasts. FEBS Lett. 1980, 121, 78–80. 10.1016/0014-5793(80)81270-1.

[ref105] Krewald V.; Retegan M.; Cox N.; Messinger J.; Lubitz W.; DeBeer S.; Neese F.; Pantazis D. A. Metal oxidation states in biological water splitting. Chem. Sci. 2015, 6, 1676–1695. 10.1039/C4SC03720K. 29308133 PMC5639794

[ref106] Yang K. R.; Lakshmi K. V.; Brudvig G. W.; Batista V. S. Is Deprotonation of the Oxygen-Evolving Complex of Photosystem II during the S_1_ → S_2_ Transition Suppressed by Proton Quantum Delocalization?. J. Am. Chem. Soc. 2021, 143, 8324–8332. 10.1021/jacs.1c00633. 34029102

[ref107] Saito K.; Nishio S.; Asada M.; Mino H.; Ishikita H. Insights into the protonation state and spin structure for the *g* = 2 multiline electron paramagnetic resonance signal of the oxygen-evolving complex. PNAS Nexus 2023, 2 (8), pgad244 10.1093/pnasnexus/pgad244. 37564363 PMC10411963

[ref108] Assunção R.; Zaharieva I.; Dau H. Ammonia as a substrate-water analogue in photosynthetic water oxidation: Influence on activation barrier of the O_2_-formation step. Biochim. Biophys. Acta, Bioenerg. 2019, 1860, 533–540. 10.1016/j.bbabio.2019.04.005. 31034801

