# Supplementary material for: Ammonia Binding to the Oxygen-Evolving Complex Probed by High-Energy Resolution Fluorescence Detected X-Ray Absorption Spectroscopy
Source: J Phys Chem B. 2025 Apr 3;129(15):3776–87. doi: 10.1021/acs.jpcb.5c00269 (PMC12010325; doi:10.1021/acs.jpcb.5c00269)
Supplement: Supplementary file 1 — jp5c00269_si_001.pdf [file jp5c00269_si_001.pdf]

SUPPORTING INFORMATION

for

Ammonia Binding to the Oxygen Evolving Complex Probed by High-Energy Resolution Fluorescence Detected X-ray Absorption Spectroscopy

Maria Chrysina,[a,b] *Maria Drosou,*[c] *Dimitrios A. Pantazis,*[*c] *Serena DeBeer*[* a]

[a] Max Planck Institute for Chemical Energy Conversion, Stiftstr. 34-36, Mülheim an der Ruhr 45470, Germany

[b] Institute of Nanoscience & Nanotechnology, NCSR "Demokritos", Athens 15310, Greece

[c] Max-Planck-Institut für Kohlenforschung, Kaiser-Wilhelm-Platz 1, 45470 Mülheim an der Ruhr, Germany

* Email: serena.debeer@cec.mpg.de, dimitrios.pantazis@kofo.mpg.de

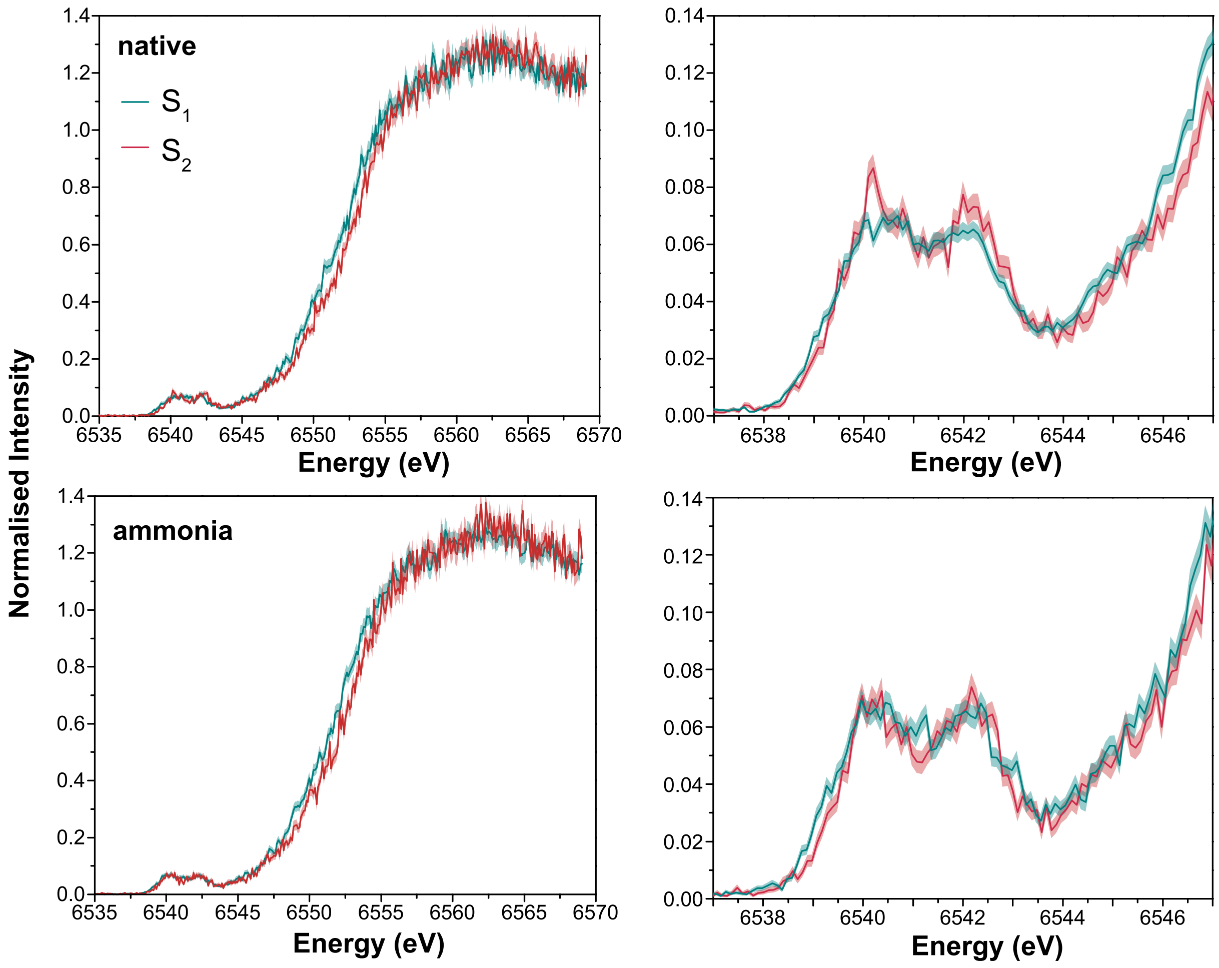

Figure S1. Mn K-edge HERFD XAS spectra of the S_1 and S_2 states of the native (up) and ammonia-treated (down) Photosystem II. The shaded areas represent the standard error. On the left, the XANES scans are presented, while on the right, the pre-edge scans. The XANES scans are normalized by area to the long (6530-6800 eV) scans. The pre-edge scans (6537-6547 eV) are normalized by area to the XANES scans.

The standard error was calculated as $\sigma_{\bar{x}} = \frac{\sigma}{\sqrt{n}}$ where $\sigma = \sqrt{\frac{\sum_{i=1}^{n}(x_i - \bar{x})^2}{n-1}}$ is the standard deviation, $\bar{x}$ is the mean of scans x_1–x_n, n: total number of scans.

The experimental spectra are oversampled (energy step = 0.1 eV), especially at the edge and post-edge region and hence, were binned. In the XANES scans, an 11-point filter was applied (Figure 2b, main text), while in the pre-edge scans, a 5-point filter was applied (Figure 2c and Figure S2).

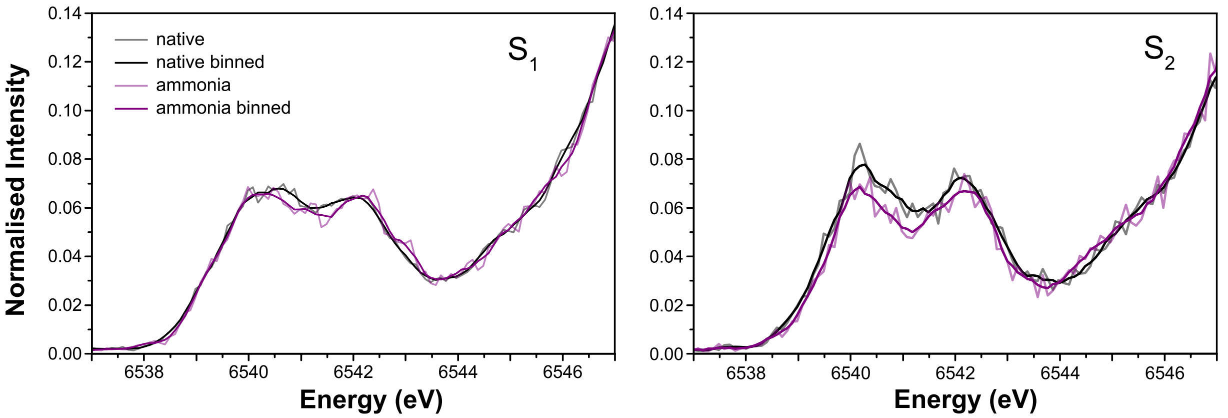

Figure S2. Raw Mn K-edge HERFD XAS spectra of the native (in black) and ammonia-treated (in purple) S_1 (left) and S_2 state (right) in the pre-edge region compared with the 5-point binned spectra.

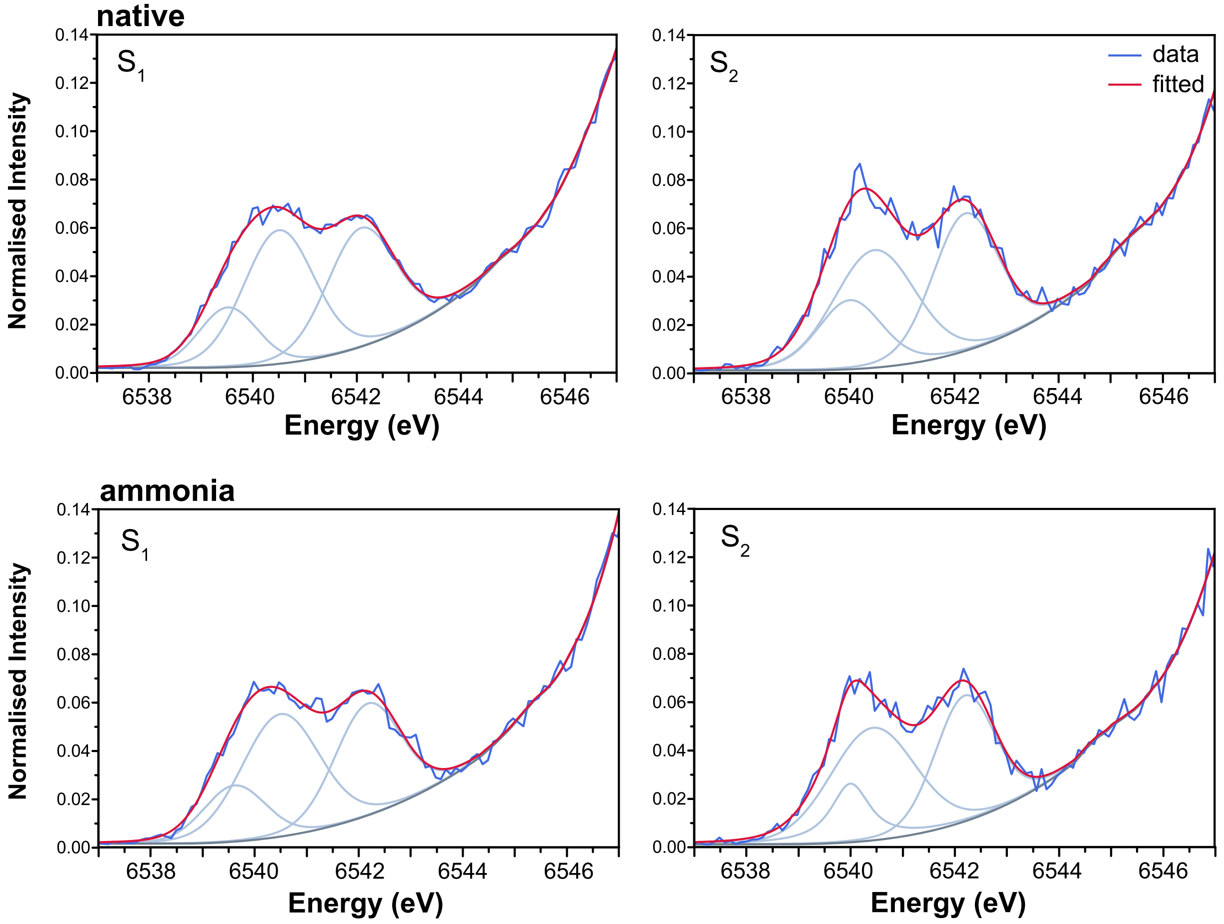

Figure S3. Fitting of raw Mn K-edge HERFD pre-edge spectra with Voigt curves after subtracting a polynomial spline. The Voigt curves used for each spectrum fitted are listed in Table S1.

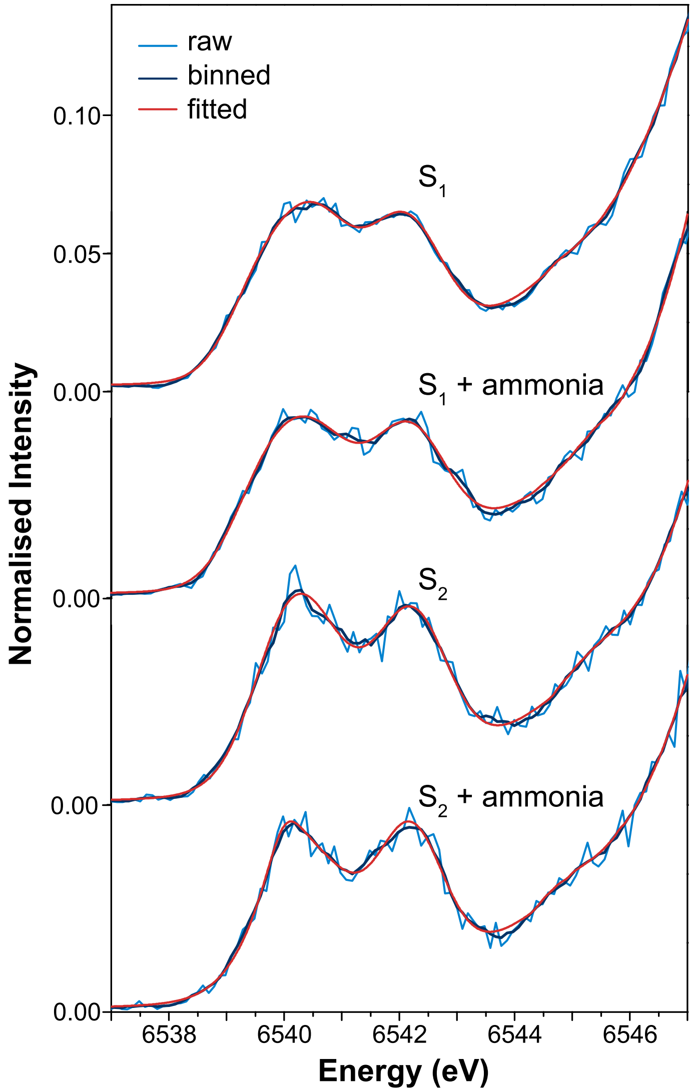

Figure S4. Comparison of the raw, 5-point binned and fitted pre-edge Mn K-edge HERFD XAS spectra for S_1 and S_2 of native and ammonia-treated Photosystem II.

Table S1. Parameters of Voigt curves used for fitting the pre-edge.

Optimized parameters	Native S_1	Native S_2	Ammonia S_1	Ammonia S_2
Amplitude 1	0.04	0.05	0.04	0.03
Center 1	6539.52	6540.00	6539.62	6540.00
Sigma 1 (Gaussian width, eV)	0.52	0.53	0.55	0.10
Gamma 1 (Lorentzian width, eV)	0.10	0.19	0.10	0.42
Amplitude 2	0.10	0.10	0.10	0.10
Center 2	6540.50	6540.48	6540.50	6540.44
Sigma 2 (Gaussian width, eV)	0.64	0.74	0.70	0.77
Gamma 2 (Lorentzian width, eV)	0.10	0.10	0.10	0.10
Amplitude 3	0.09	0.10	0.08	0.08
Center 3	6542.08	6542.21	6542.17	6542.20
Sigma 3 (Gaussian width, eV)	0.62	0.59	0.60	0.53
Gamma 3 (Lorentzian width, eV)	0.10	0.10	0.10	0.10
AREA	0.22	0.241	0.21	0.21

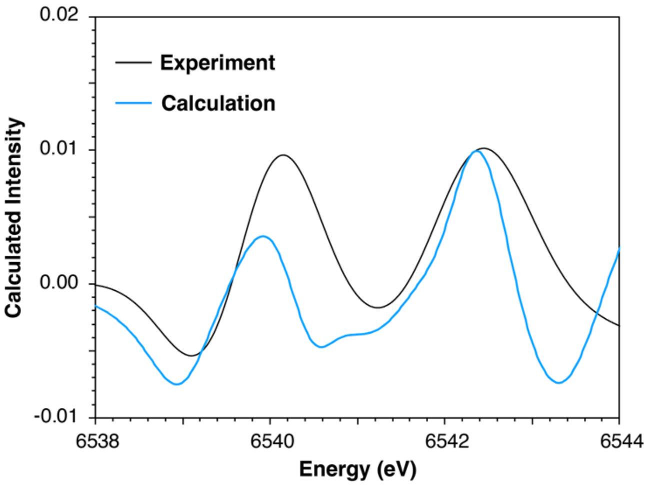

Figure S5. Experimental (black) and calculated (blue) Mn XAS $S_2 - S_1$ difference spectra. The RMSD of the calculated curve from the experimental data is 0.0055.

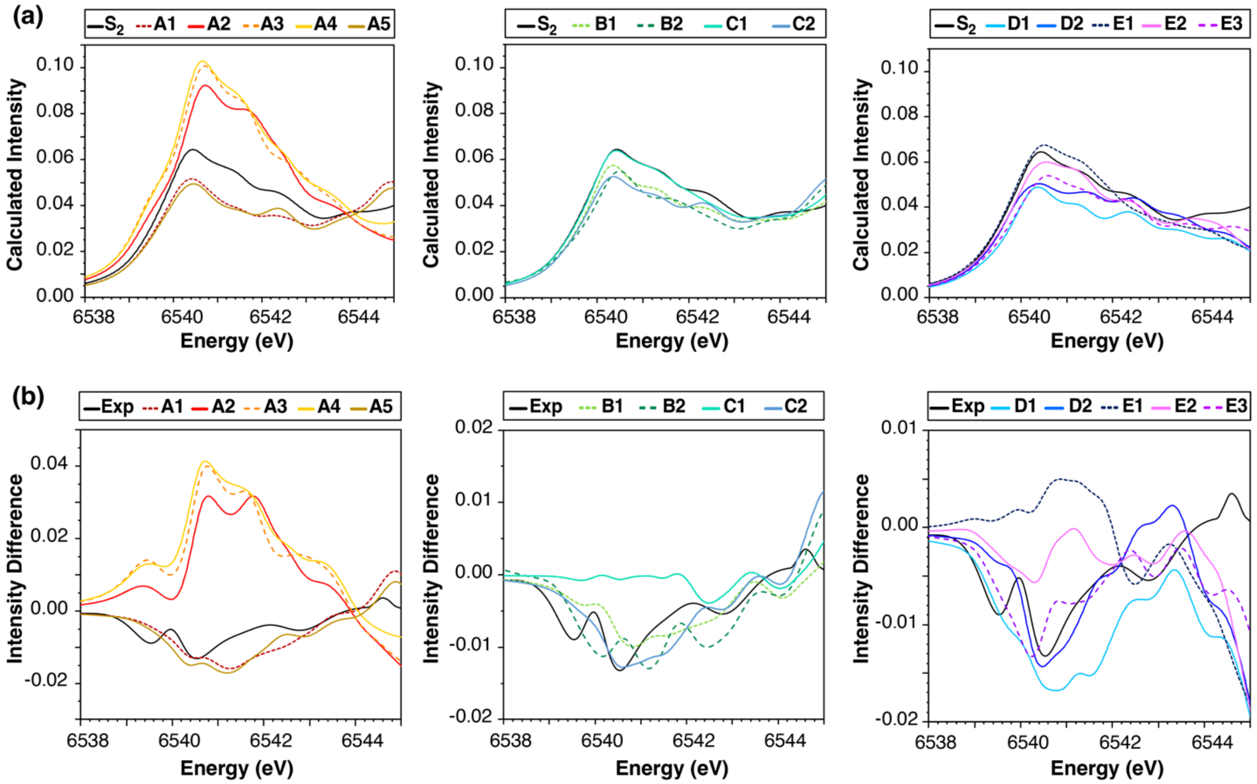

Figure S6. (a) Calculated Mn K-edge XAS spectra of the S_2 state model with W2 = OH (black) compared to the $S_2^{NH_3}$ models separated in groups A (left), B and C (middle), and D and E (right). (b) Experimental (black) and calculated difference Mn K-edge XAS spectra of the ammonia-treated S_2 state from the untreated S_2 state, i.e. $S_2^{NH_3} - S_2$.

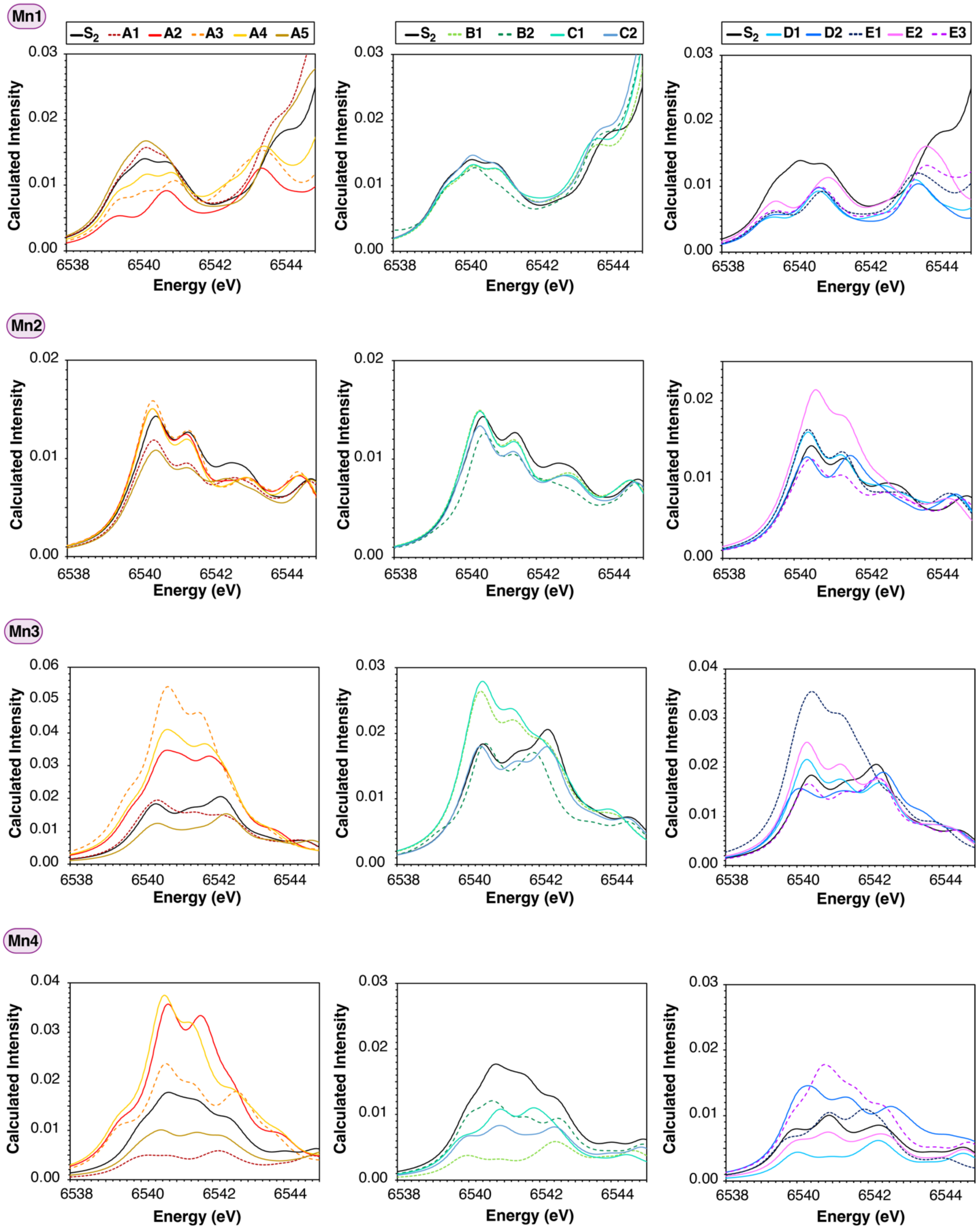

Figure S7. Calculated XAS spectra for individual Mn ions of S_2-NH_3 models compared to the S_2 state (W2 = H_2O).

Table S2. RMSDs of the calculated from the experimental $S_2^{NH_3} - S_2$ difference spectra in the region between 6538.0 and 6544.5 eV, using the S_2 (W2 = OH) and S_2 (W2 = H_2O) models.

	S_2 (W2 = OH)	S_2 (W2 = H_2O)
**A1**	0.00404	0.00517
**A2**	0.02184	0.02165
**A3**	0.02509	0.02514
**A4**	0.02653	0.02593
**A5**	0.00416	0.00491
**B1**	0.00276	0.00422
**B2**	0.00313	0.00415
**C1**	0.00571	0.00645
**C2**	**0.00223**	**0.00338**
**D1**	0.00612	0.00665
**D2**	0.00432	0.00469
**E1**	0.00860	0.00926
**E2**	0.00481	0.00549
**E3**	0.00340	0.00362

Orca input file for XAS calculations:

```
! UKS TPSSh RIJCOSX ZORA ZORA-def2-TZVP(-f) SARC/J CPCM
! NoTrah TightSCF

%basis newgto C "ZORA-def2-SVP" end
       newgto H "ZORA-def2-SVP" end
end

%cpcm
    surfacetype vdw_gaussian
    epsilon 6.0
end

%scf maxiter 200
     shift shift 0.10 erroff 0.1 end
     FlipSpin 1,2 FinalMs 0.5
end

% tddft NRoots 150
        MaxDim 8
        OrbWin[0]=0,0,-1,-1
        OrbWin[1]=0,0,-1,-1
        DoQuad true
end

*xyzfile 1 14 filename.xyz
```
